# Identification of Antibacterial Cyclic Peptides with a High-Throughput Cell-Based Dropout Screen

**DOI:** 10.1021/jacs.6c09188

**Published:** 2026-07-25

**Authors:** Leonie M. Windeln, Lewis W. Mitchell, Agnieszka B. Wisniewska, Alexander McDermott, Scott S. Walker, Abbas M. Walji, Ali Tavassoli

**Affiliations:** † School of Chemistry and Chemical Engineering, 7423 University of Southampton, Southampton SO17 1BJ, United Kingdom; ‡ 2793 Merck & Co. Inc., West Point, Pennsylvania 19486, United States

## Abstract

Antimicrobial resistance is a growing global health threat, necessitating new antibiotics and discovery strategies. Here, we report a target-agnostic negative-selection screening platform that combines an intracellular library of 3.2 million cyclic peptides with next-generation sequencing to identify antibacterial cyclic peptides through the depletion of their encoding sequences. Clustering of depleted sequences by shared pharmacophores enables robust hit identification and allows focused library design. Using this approach, we identify a cyclic peptide scaffold that inhibits the previously untargeted essential iron–sulfur cluster carrier protein ErpA. This work establishes a general and scalable framework for the discovery of intracellular antibacterial targets and associated inhibitors directly in living cells.

## Graphical abstract

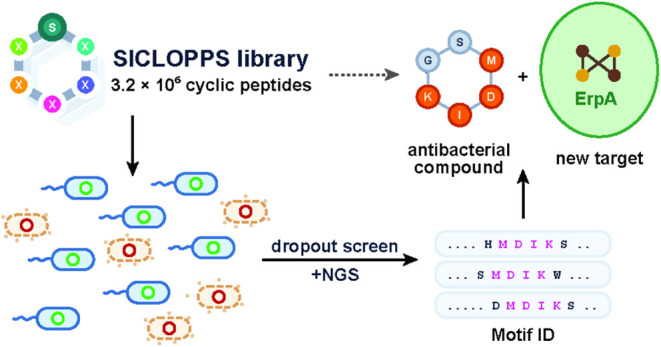

## Introduction

The rise of antimicrobial resistance, coupled with a slowdown in the discovery of new antibiotic classes since the 1990s, represents a major threat to global health.^1^ This is especially true for Gram-negative pathogens such as *Escherichia coli*, *Klebsiella pneumoniae*, *Pseudomonas aeruginosa*, and *Acinetobacter baumannii* as they have acquired resistance against most current antibiotics.^2^ Natural products have historically provided a rich source of antibiotics, and recent efforts have focused on mining previously uncultured microorganisms to expand accessible chemical diversity.^3^ Cyclic peptides have also emerged as a valuable class of antibacterial agents;^4–6^ their conformational diversity allows the targeting of challenging targets, including protein–protein interactions that have traditionally proven elusive to inhibition with classic modalities.^7–11^ Another key advantage of cyclic peptides is the availability of several methods for the rapid production and screening of large, genetically encoded libraries,^12^ which has proven to be a powerful tool in drug discovery.^13,14^ Whilst most of these platforms use affinity-based *in vitro* selections against recombinant proteins, one approach combines the intracellular production of millions of cyclic peptides with functional cell-based assays. Split-intein circular ligation of peptides and proteins (SICLOPPS) is a genetically-encoded method for the generation of head-to-tail cyclic peptide libraries in living cells (Figure S1).^15^ In this system, each library member is DNA-encoded such that individual cells produce a single member of the cyclic peptide library following intein-mediated protein splicing. This “one cell-one compound” format enables direct functional screening of cyclic peptide libraries of millions of members within intracellular environments, providing a powerful platform for ligand discovery. Importantly, intracellular expression enables interrogation of intracellular targets in their native environment without requiring compound uptake across the cell membrane during the hit discovery phase of drug discovery. SICLOPPS libraries have been successfully used for screens in a variety of systems (*E. coli*, yeast, and mammalian cells) to enable the discovery of inhibitors against a wide range of targets.^10,12,16^

We recently incorporated next-generation sequencing (NGS) into the SICLOPPS screening workflow to accelerate and simplify the screening process, and reduce false positives.^17^ Here, we introduce a sequencing-based negative-selection screening strategy, representing a conceptual departure from the positive-selection approaches that dominate most drug discovery workflows. In this dropout screen, library members whose intracellular expression reduces host cell fitness or viability are identified by NGS based on depletion of their encoding sequences in the surviving population relative to the parent library (Figure 1A). Because this phenotypic strategy does not require a predefined target, it also provides a potential route to identifying new antibacterial protein targets.

**Figure 1:**
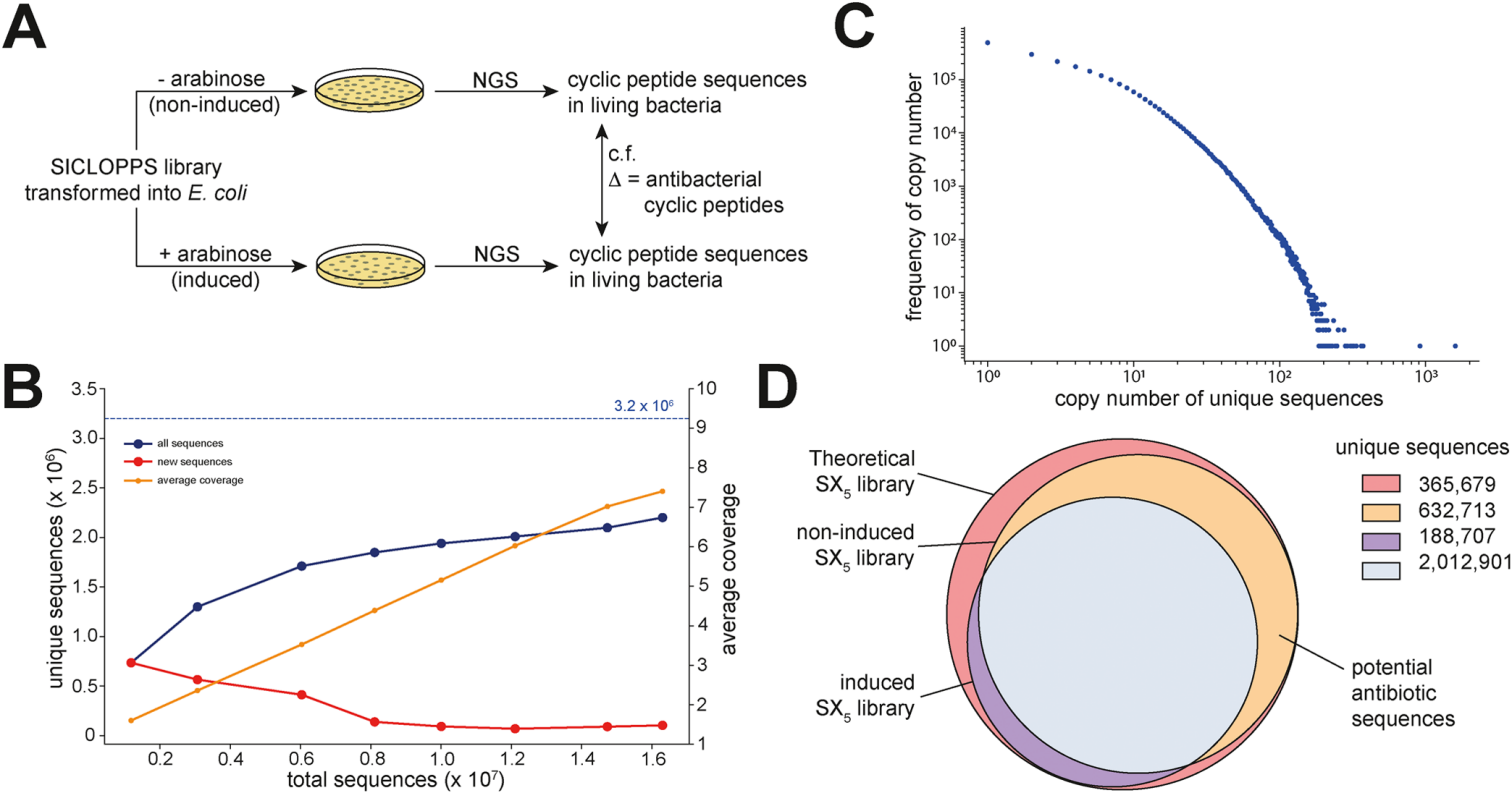
Dropout screen overview and library coverage. **A)** In the non-induced library, all correctly cloned inteins can be recovered, as no expression takes place. Inducing expression of inteins on agar plates (after transformation) results in the production of cyclic peptides *in vivo*. Colonies containing plasmids that are coding for nontoxic cyclic peptides are formed, whilst those coding for toxic cyclic peptides kill the host cell and get lost. **B)** Sequence coverage of the library in relation to the total sequences acquired. Each data point represents a different sequencing run. In orange, the calculated average coverage (total sequences/unique sequences). **C)** Coverage plot of all sequences after induction. Each unique sequence is assigned a copy number in the library. The distribution of these copy numbers within the library is then plotted as a frequency. **D)** Venn diagram of unique sequences in the SX_5_ screen, the simulated library comprises all possible 3.2 × 10^6^ unique sequences. The plasmid and induced libraries were experimentally determined.

To demonstrate this concept, we applied the approach to a SICLOPPS library encoding 3.2 × 10^6^ unique *cyclo-*SXXXXX (SX_5_) cyclic hexapeptides (where X = any canonical amino acid) in *E. coli*. As incomplete sequencing coverage can generate apparent dropout events unrelated to biological activity, we developed a computational framework that clusters depleted sequences around shared pharmacophore motifs to distinguish true hits from sampling noise. This analysis enabled the construction of a focused library enriched for active motifs, leading to the identification of a cyclic peptide scaffold and its intracellular target, the essential iron–sulfur cluster carrier protein ErpA.

Together, these results establish intracellular negative-selection dropout screening as a scalable strategy for the discovery of antibacterial targets directly in living cells, as well as providing an inhibitor that can serve as the starting point for the development of new antibiotics.

## Results

### Library Preparation and Intracellular Induction

A genetically encoded SICLOPPS SX_5_ library of 3.2 × 10^6^ members was generated using standard molecular biology techniques for use in our dropout screen.^18^ This plasmid library was deep sequenced prior to transformation into *E. coli* in order to identify and quantify the SICLOPPS plasmids present in the library. These sequences were the starting point for the dropout screen; upon transformation into a bacterial host intein expression and subsequent cyclic peptide production is induced with arabinose. Cyclic peptides with bacteriostatic or bactericidal properties will result in a reduction in growth or death of the *E. coli* host, leading to a decrease in copy number (or loss) of the corresponding SICLOPPS plasmid. Thus, isolating SICLOPPS plasmids from surviving *E. coli* followed by deep sequencing has the potential to identify missing or under-represented sequences (c.f. parent library).

*E. coli* K-12 was transformed with plasmids encoding the SX_5_ library with a transformation efficiency of 1.96 × 10^8^ colony forming units/μg DNA. After recovery, cells were spread and incubated on rich medium with and without arabinose (0.002% to induce SICLOPPS cyclic peptide production) for 18 h, and deep sequenced using a previously reported method specific to SICLOPPS libraries.^17^ Stringent filtering was applied to the NGS reads to minimize false positives, including base call accuracy of >99.9% (Q30), the presence of a known restriction digest site (NheI, used for cloning the library), and fidelity of the first three amino acids of the N-terminal intein which forms the splice junction. The limited sequence diversity within the amplicon (15 variable bases) imposes constraints on sequencing coverage and complete recovery of sequences encoding all theoretical library members (3.2 × 10^6^). We therefore conducted multiple deep sequencing runs to ensure sufficient coverage of our library; for the pre-induced library, we observed 2.3 × 10^7^ total and 2.6 × 10^6^ unique cyclic peptides after filtering and pooling of 11 sequencing runs (Figure S2A). Post-induction, we observed 1.62 × 10^7^ total sequences and 2.2 × 10^6^ unique sequences from 8 sequencing runs (Figure 1B). We also examined the contribution of each sequencing run for the induced library to new sequences added to the total sequences. The data shows that the total number of sequences converges to a plateau after the fourth sequencing run; after this, each additional sequencing run only added relatively few new sequences (Figure 1B and Figure S2A). Although the coverage per sequence was 5–8 fold, the theoretical maximum number of 3.2 × 10^6^ unique sequences was not observed in either case. The main reason for this is the inherent limitations of sequencing a low-diversity library that contains only 15 variable bases in each amplicon (i.e., the region encoding the 5 randomized amino acids in the cyclic hexapeptide). There may also be sequences that have a negative impact on *E. coli* growth through low level, non-induced expression. We also examined the copy number distribution of each sequence within the induced library, and observed balanced library composition with most cyclic peptide sequences present 1–5 times which represents <0.007% of the library each (Figure 1C and Figure S2B).

There were 2,012,901 sequences present in the induced library that were also found in the non-induced library (i.e., not toxic to the host), with an additional 188,707 sequences observed after induction but not before (Figure 1D). These are most likely a consequence of the incomplete sequencing of the plasmid library, and they are likely to also be present in the non-induced library. There were 632,713 sequences which were present in the plasmid library but not in the induced library; these are the dropout sequences with potential antibiotic properties. However, it is unlikely that so many members of the library are toxic to the bacterial host. As before, most of these sequences are likely not read due to sequencing limitations. Nonetheless, true dropout sequences will be present amongst these sequences, so we turned our efforts to developing a computational method for identifying these sequences from this population.

### Identifying True Dropout Sequences

Previous work has shown that a 2–4 amino acid pharmacophore is crucial for biologic activity of SICLOPPS-derived cyclic hexapeptides.^8,19^ We hypothesized that the active pharmacophore of any antibiotic in our screen will be shared by more than one cyclic peptide and therefore sought to collate the dropout sequences by shared pharmacophores (rather than the whole sequence of the hexamer). Current motif searching algorithms are often deployed on enriched libraries from phage display or mRNA display screens,^20^ with around 1,000 linear peptide sequences as the input for alignment and pattern identification in enriched cyclic peptides.^21,22^ Analysis of our dataset would require identification of dropout (rather than enriched) sequences from a substantially larger dataset. Another challenge was that unlike other genetically encoded cyclic peptide libraries, SICLOPPS-derived cyclic peptides are head-to-tail cyclized with an amide bond, so the point of cyclization is indistinguishable after intein splicing. Thus, every position in the cyclic peptide, including the first and last amino acid, may be required for target binding, which needs to be accounted for in the computational analysis.

To address these challenges, we developed a matrix-based approach to data analysis that was similar to neural network convolution. The axis length of these matrices was 20, representing each of the 20 canonical amino acids, and the number of axes was defined by the length of the pattern. We created 2-, 3-, and 4-dimensional matrices that counted the absolute occurrence of all possible di-, tri-, and tetra-peptides in a SICLOPPS SX_5_ library. Additional matrices were created for the induced and non-induced library, and the changes in abundance from non-induced to induced was calculated for each di-, tri-, and tetra-peptide motif as a % drop. Motifs that were present in fewer cyclic peptides after induction would have a greater % decrease than motifs found more frequently, and the drop would give an indication of toxicity of the respective peptide motif in the context of a cyclic hexapeptide. We ranked motifs across the datasets based on reduction in copy number upon library induction. We observed the largest change in copy number upon induction for tetrapeptide sequences (Figure S3) and therefore focused further analysis on tetrapeptide motifs (Table 1). Each tetrapeptide motif has a theoretical maximum copy number of 40 for motifs that do not include a serine (allowing for one random position either before or after the motif). None of the most dropped out motifs included a serine and throughout the most depleted sequences, polar and charged amino acids were more abundant than aliphatic ones. At least one, but often multiple histidine (H), asparagine (N), glutamine (Q), or lysine (K) were present in each of the top motifs (Table 1 and Figure 2). The biological relevance of HHHH as the most dropped out motif is questionable, as poly-His-tags are frequently over-expressed in *E. coli* strains without indication for toxicity. We rationalized such false positives as being due to the low codon degeneracy of amino acids such as histidine in the NNS library resulting in the presence of only one codon, leading to oversensitivity in our assay to those sequences being missed e.g., due to the NGS limitations described earlier. Thus, such low prevalence sequences will dropout easier than those that start with a higher copy number.

**Table 1:** Top 15 Most Dropped-Out Tetrapeptide Motifs Identified in Screen

Tetrapeptide motif	% Drop	% Drop from plasmid	Induced library	Plasmid library
HHHH	100	100	0	10
NNHI	98	93	1	15
YQNN	98	93	1	14
DMKK	98	92	1	13
DENH	98	92	1	13
HECD	98	92	1	12
CKHC	95	90	2	20
KQNQ	98	89	1	9
KQQQ	98	89	1	9
HKMN	95	89	2	18
MDIK	95	88	2	16
CQHD	95	87	2	15
HQDD	95	87	2	15
QNNN	95	86	2	14
TNHD	95	86	2	14

**Figure 2:**
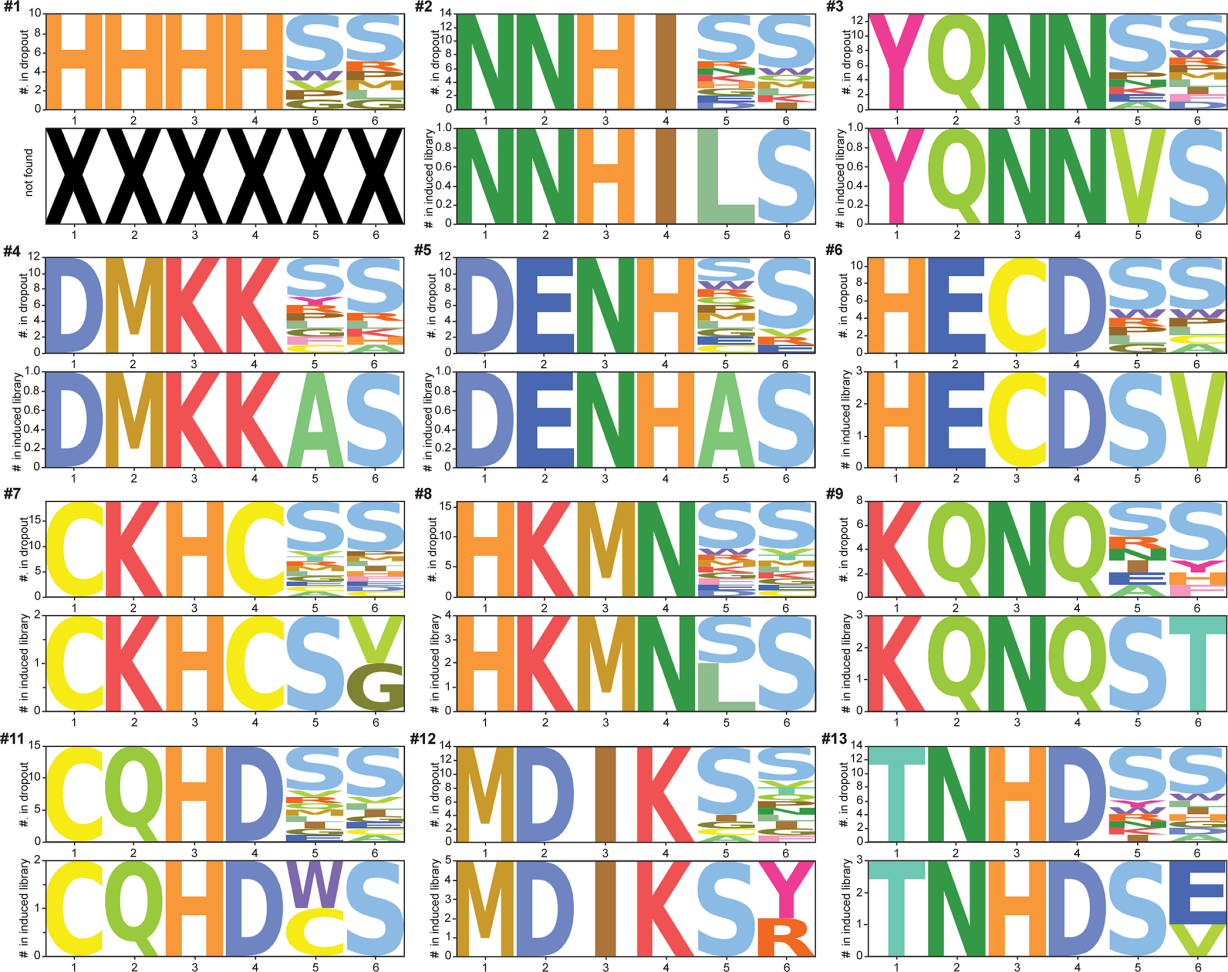
Sequence logo of most dropped-out cyclic peptides in the post-induction samples versus the parent library. Sequences show the number of cyclic peptides with each motif lost upon induction (top) and those with the same motif present in the induced library (bottom). The numbers in bold represent the rank of each motif in the drop-out screen analysis; the 10th motif KQQQ (not shown) was excluded from further analysis due to similarity to the 9th motif KQNQ. Sequence logos were created with Logomaker.^23^

### Focused Libraries of Tetrapeptide Motifs

The top dropout sequences were further evaluated using a focused library comprising the 12 most enriched tetrapeptide motifs. In this library, motifs were positioned either directly after the invariant serine residue (S–motif–X) or separated from it by a randomized amino acid (S–X–motif), based on their observed positional context in the dropout sequences. For example, the MDIK motif was only observed in the S–X–motif format (e.g., SXMDIK), and not as SMDIKX, suggesting the latter is potentially toxic to the host bacterium (Figure 2).

Thirteen focused sub-libraries were generated using the same protocol as for the initial SICLOPPS library. These libraries were pooled and used to transform *E. coli*, plated on LB agar plates without and with arabinose (0.002%, to induce SICLOPPS cyclic peptide production). All colonies from both conditions were collected and analyzed by NGS, yielding a total of 30,000 cyclic peptide sequences for the induced library and 70,000 sequences for the non-induced library. The datasets were normalized to enable identification of sequences depleted post-induction.

The focused libraries containing HHHH, HECD, HKMN, DMKK, CQHD, and MDIK showed a drop in prevalence after induction, with the overall prevalence of each motif varying from 0.3% to 15.4% (Table 2). Each focused library was added to the pool in equal amounts; we therefore expected their ratio to be similar in the pre-induction pool (∼7.7%), but this was not the case. The SHHHHX library was the most abundant both before (15.4%) and after induction (13.8%), followed by SHKMNX with 14.4% before and 13.8% after induction. Conversely, the SMDIKX library constituted only 0.3% of the total sequences pre and 0.2% post induction and was the least abundant in the focused library pool. Second and third lowest were the SCKHCX (2.6% to 2.8%) and SXYQNN (3.6% to 4.1%) libraries.

**Table 2:** Prevalence and Fold Change of Focused Library Dropout Screen, a Dash Indicates That No Missing Sequences Were Found

	Non-induced library		Induced library			
Library motif	Prevalence [%]	Missing sequences	Prevalence [%]	Missing sequences	Fold change	Fold Dropout
SMDIKX	0.3	X = V, P, Y, D, E, N, H, K	0.2	X = V, P, Y, D, E, N, H, K, L, T, Q	0.66	1.78
SHHHHX	15.4	-	13.8	-	0.88	1.13
SHECDX	11.8	-	10.8	-	0.91	1.1
SXCQHD	7	-	6.4	-	0.91	1.1
SXHHHH	5.2	X = N	4.8	X = N	0.92	1.08
SHKMNX	14.4	-	13.8	-	0.95	1.06
SDMKKX	6.1	-	6	-	0.96	1.04
STNHDX	7.8	-	8	-	1	1
SCKHCX	2.6	X = Y	2.8	X = M	1.08	0.93
SKQNQX	8.2	-	9.1	-	1.09	0.92
SXDENH	10.4	-	11.6	-	1.1	0.91
SXYQNN	3.6	-	4.1	-	1.13	0.89
SXNNHI	7.3	-	8.6	-	1.16	0.86

We reasoned that potential leaky expression from the arabinose promoter may result in the low-level transcription and translation of the SICLOPPS construct, yielding small amounts of cyclic peptide, even in uninduced cells, which may provide a rationale for sequences being lost without induction, due to their high toxicity to the host. According to this hypothesis, our most promising antibiotic leads would be found in the least abundant motif library, which was SMDIKX at 0.3% pre- and 0.2% post induction. This sub-library also showed the largest dropout ratio upon induction, with only 12 out of 20 possible sequences found before induction and 9 out of 20 after induction (Figure 3A).

**Figure 3:**
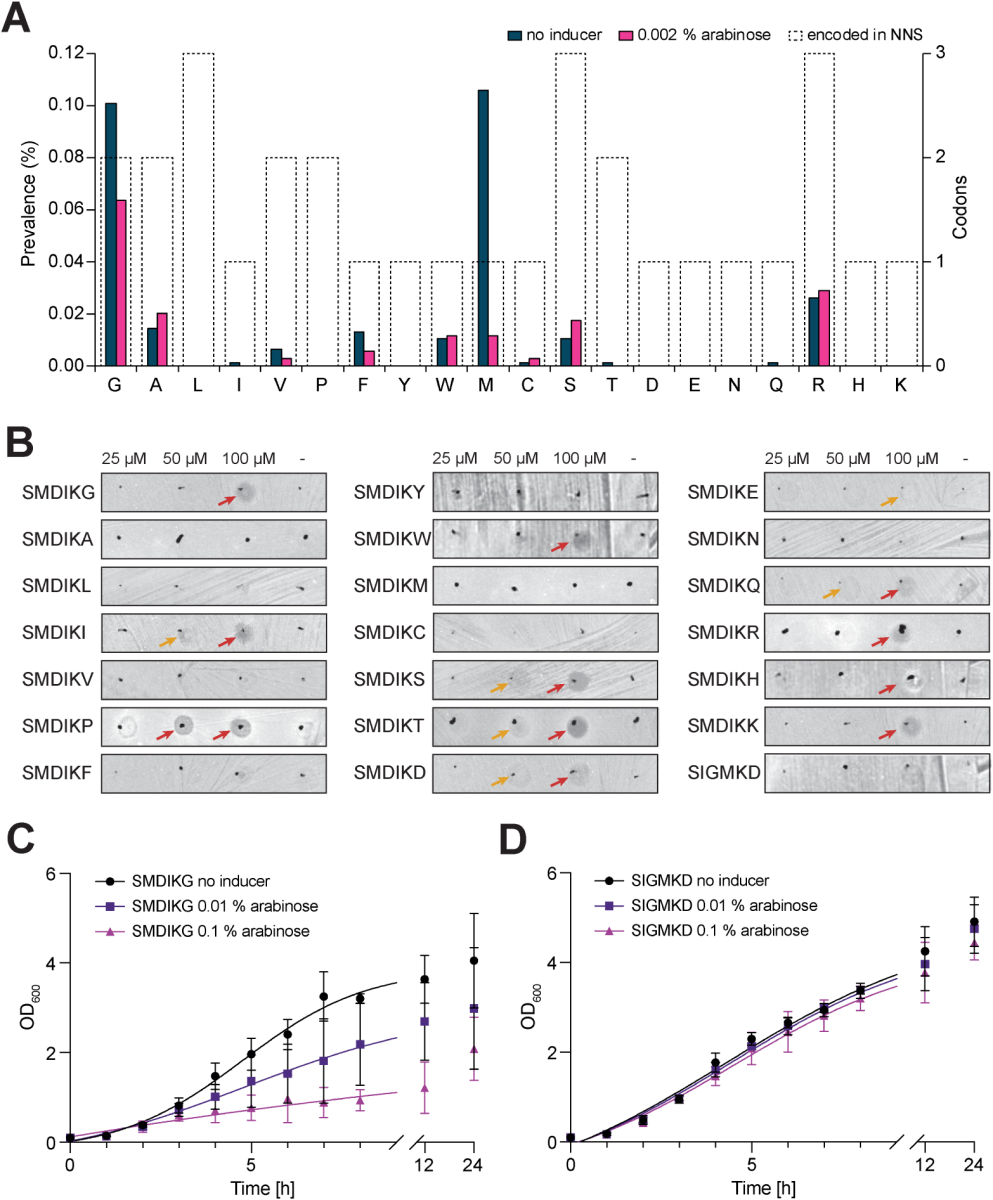
Assessing the activity of cyclo-SMDIKX in *E. coli*. **A)** Fraction of each library member in the SMDIKX focused sub-library; blue bars show fraction within the non-induced library after NGS, pink bars show fraction within the induced focused library after NGS, boxes around the prevalence bars indicate how many codons encode for each amino acid in the library. **B)** ZOI assay for *cyclo-*SMDIKX derivatives in *E. coli* JW3596 **C)** Inducer-dependent growth of *E. coli* DH10β harboring a plasmid encoding cyclo-SMDIKG. **D)** Inducer-dependent growth of *E. coli* DH10β harboring a plasmid encoding the scramble control cyclo-SIGMKD. All data is n = 3 ± SEM.

Examining each remaining amino acid in the random position of *cyclo-*SMDIKX library, the biggest change from pre- to post-induction was the drop in methionine prevalence by 10-fold (from 0.11% to 0.01%). A similar effect could be observed for *cyclo-*SMDIKG, which was 60% less present in the induced library than in the non-induced library (Figure 3A). Overall, the focused library dropout experiment suggests that 15 members of the *cyclo-*SMDIKX library have a negative impact on growth of the host bacterium.

To assess the false-discovery rate of the motif-dropout analysis, we used the sequence encoded by the library plasmid template *cyclo*-SAAAAA (SA_5_), present at anomalously high copy number (1,649 copies; the single most abundant sequence in the pre-induction library), as an endogenous internal control. This sequence is not expected to drop out upon induction, and any artifactual depletion of abundant sequences would be detectable in its behavior. At the dipeptide level, motifs derived from the control sequence ranked in the bottom quartile (Table S1). In contrast, the dipeptide motifs of *cyclo*-SMDIKG revealed a clear gradient of dropout signal; the MDIK-core dipeptides MD, DI, IK, DM, ID, and KI all ranked in the top 100, while the flanking S- and G-containing dipeptides SM, MS, KG, and GK ranked mid-table, and GS and SG ranked near the SA_5_ floor. This gradient independently corroborates MDIK as the active pharmacophore and S and G as passive positions. At the tripeptide level, pure polyalanine tripeptides were undetected. Of the six possible tripeptide motifs within *cyclo-*SMDIKG, only MDI and DIK were detectable, ranking 662nd and 504th out of 1,600 (Table S2) further supporting the MDIK as the bioactive motif. At the tetrapeptide level, alanine-containing motifs were entirely absent from the most-dropped motifs, while MDIK emerged as a clear top-ranked hit. The requirement for tetrapeptide resolution to detect MDIK confirms that the four-residue pharmacophore length is a genuine property of the active motif, and that the absence of signal from the internal control reflects biological activity rather than sampling noise.

To assess and further verify the ability of individual *cyclo-*SMDIKX library members to inhibit bacterial growth, we attempted to generate SICLOPPS plasmids encoding each of the 20 cyclic peptides in this focused library. We were unable to generate plasmids encoding the majority of *cyclo-*SMDIKX variants (X = A, L, P, Y, D, E, N, H, K, I, T, and Q) despite repeated attempts. Sequencing revealed frequent mutations disrupting the SICLOPPS construct, consistent with strong selection against intact expression of these cyclic peptides (Figure S4). This included point mutations in the N-intein (I_N_) resulting in a defective splice junction, deletions in the I_N_ between 3–10 bp downstream of the extein leading to truncated inteins. Mismatch ligations, where the C-intein was ligated to parts of the plasmid downstream of the N-intein, and original (SA_5_) vector retention accounted for 30% of the analyzed samples, whilst such sequences made up only 1% of the original SX_5_ library. For some samples, no sequencing results could be obtained, with Sanger sequencing output file of shorter than 200 bp. The sequences that were absent from the pre- and/or post-induction samples from the focused dropout screen of SMDIKX could also not be cloned as a plasmid here. Given the proposed antibacterial properties of the SMDIKX family, the leaky expression under non-inducing conditions of the inteins was thought to be causing cell death in cases of correctly cloned plasmid, leading to only the cells with mutated nonfunctional SICLOPPS plasmids to form colonies. Even for cyclic peptides where an encoding plasmid could be obtained (X = G and M), a large portion of the plasmids were found to contain mutations in the SICLOPPS constructs. Together, these observations provide further evidence that the *cyclo-*SMDIKX family are toxic to their host bacterium.

To directly test the effect of the *cyclo-*SMDIKX family on *E. coli* growth we tested synthetic samples of each peptide (25, 50, and 100 μM) in a zone of inhibition (ZOI) assay, where each compound was applied directly to freshly plated bacteria. We used the JW3596 Δ*rfaC* strain of *E. coli* in which the *rfaC* gene required for lipopolysaccharide inner core biosynthesis has been deleted, resulting in increased membrane permeability.^24^ We observed an antibiotic effect in this assay for 10 of the 20 cyclic peptides (Figure 3B), with good correlation between the cyclic peptides showing activity in this assay and the data from the focused dropout screen (Figure 3A). Most of the peptides showing activity in the zoning assay were either not present, or at very low abundance in the dropout screen (X = T, R, P, I, K, D, Q, S, W). We also synthesized *cyclo-*SIGMKD as a scrambled negative control for testing in this assay and did not observe any activity.

We chose *cyclo-*SMDIKG to take forward as a representative of this motif family for further testing. This sequence was chosen as it showed activity in the ZOI assay and had the highest abundance-change within sequences where a plasmid encoding the cyclic peptide could be generated. Using the same materials and method as for the other SMDIKX derivatives, a plasmid encoding SMDIKG was obtained, allowing testing of this molecule via SICLOPPS-production. We assessed the effect of *cyclo-*SMDIKG in liquid culture, observing arabinose-dependent reduction of growth of the host bacterium encoding this cyclic peptide. Dose-dependent inhibition was seen when *cyclo*-SMDIKG production was induced at 0.01% or 0.1% arabinose for the first 8 h (Figure 3C). Growth repression was observed for up to 12 h after induction, after which *E. coli* growth in induced samples started to recover to the levels of the uninduced sample by 24 h. This is either due to the metabolism of arabinose over time resulting in a reduction of SMDIKG expression, or the accumulation of resistance mutations in the SICLOPPS plasmid or the host bacterium. We generated a plasmid encoding *cyclo*-SIGMKD as a scrambled control and did not observe any effect from the induction of this sequence at up to 0.1% arabinose. (Figure 3D). A dose dependent effect on bacterial growth was observed on LB-agar plates containing increasing amounts of arabinose, with no growth in the presence of 0.5% arabinose (Figure S5A). We used synthetic samples of *cyclo*-SMDIKG and *cyclo*-SIGMKD (control) in an agar diffusion assay (Kirby-Bauer test) and observed a minor reduction of growth for *cyclo*-SMDIKG at 1 mg/disc (Figure S5B). The effect of this molecule was tested on the growth of wild type *E. coli* (MB4903), *E. coli* (MB4903) Δ*lpxC* with reduced LPS production leading to increased permeability, wild type *Acinetobacter baumannii* and the corresponding Δ*lpxC* strain for increased permeability. No effect was observed on the growth of the WT bacteria, whereas inhibition of growth was observed in both Δ*lpxC* strains (Figure S6). Together, this data demonstrates that *cyclo*-SMDIKG inhibits bacterial growth when expressed in *E. coli* *via* the corresponding SICLOPPS plasmid, and when administered as a synthetic sample to bacteria with a compromised cell membrane.

### Identification of Target of *Cyclo*-SMDIKG

We next sought to identify the target of *cyclo-*SMDIKG (Figure 4A). Attempts to isolate resistant mutants were dominated by mutations that reduced cyclic peptide expression via the SICLOPPS expression system, rather than genomic alterations in the host. As a result, resistance mapping did not yield a clear target assignment. We therefore turned to complementary approaches, including chemical proteomics and genetic rescue experiments, to identify the cellular target. We reasoned that the cyclic peptide could be converted to a pull-down probe through the incorporation of a linker in a position that does not interact with the target protein. To identify such a position, we conducted alanine scanning, generating 5 SICLOPPS plasmids whereby amino acids 2–6 were replaced with alanine. However, as the serine in position 1 is required for intein splicing, we could not replace it with alanine. We therefore generated a plasmid encoding *cyclo-*CMDIKG. Each plasmid was used to transform *E. coli*, SICLOPPS induced with arabinose, and the effect of the cyclic peptide on bacterial growth was measured (Figure S7). The only alanine-scanning variant retaining full activity was SMDIKA, in which the MDIK motif remained intact. A change in every other tested position led to some loss in activity, confirming the (S)­MDIK motif. *cyclo-*SADIKG, and *cyclo-*SMAIKG showed a complete loss in activity upon induction, which indicates the importance of methionine and aspartic acid within the active motif. Despite multiple attempts using our standard cloning strategy as well as ligase free cloning,^25^ a plasmid encoding *cyclo-*SMDIAG could not be obtained. As before, this is most likely due to increased toxicity (or activity) for this sequence. Surprisingly, *cyclo-*CMDIKG resulted in a decrease in growth inhibition, with more cell growth observed over time compared to *cyclo-*SMDIKG. This may be because serine plays a part in the activity of *cyclo-*SMDIKG, or it could be due to altered splice rate arising from the replacement of serine with cysteine.

**Figure 4:**
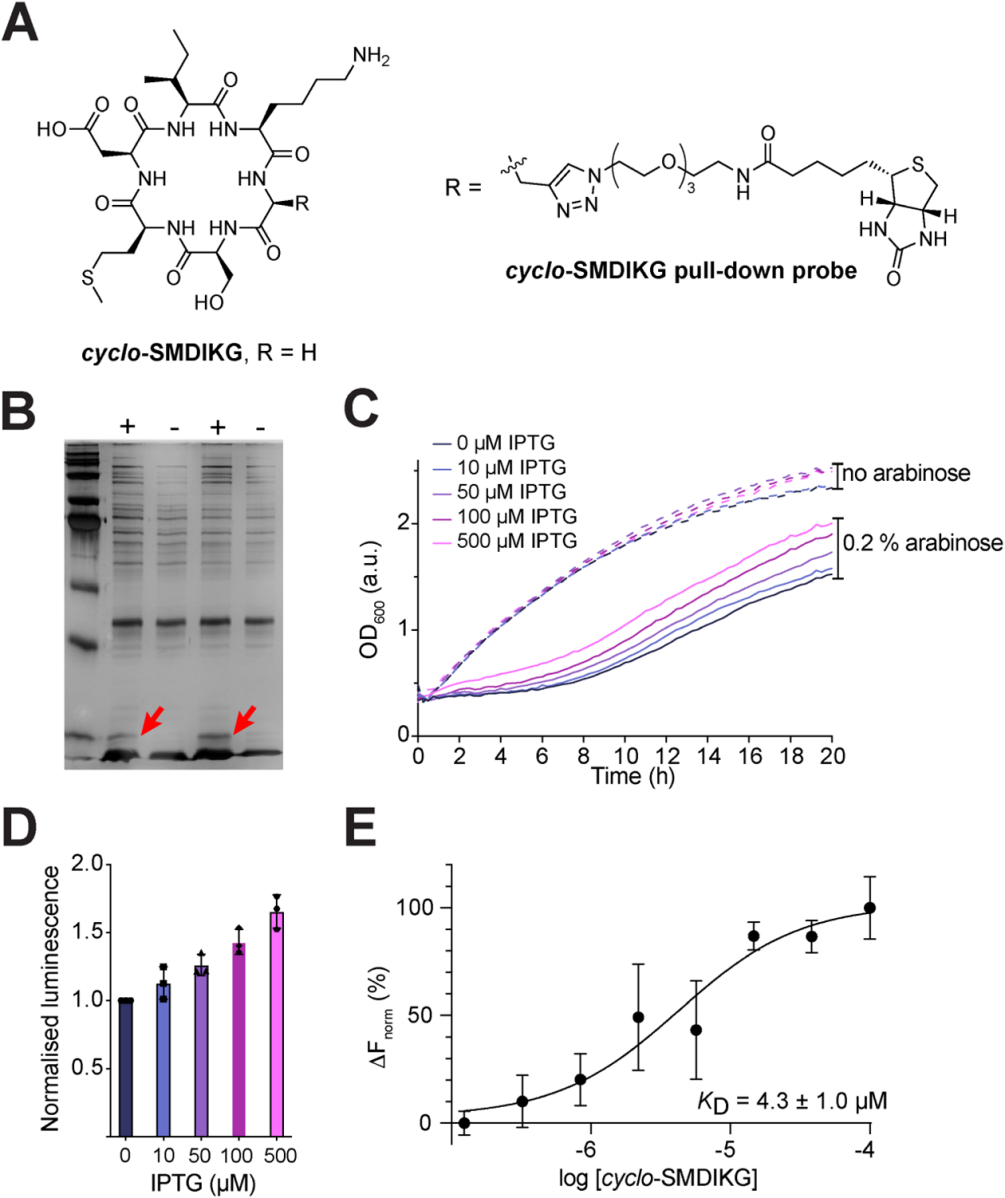
Identifying the target of *cyclo-*SMDIKG. **A)** The structure of *cyclo-*SMDIKG and the pull-down probe used in this study. **B)** SDS-PAGE showing band at 12 kDa being pulled down by *cyclo-*SMDIKG (+ lanes, red arrows). **C)** IPTG-driven over-expression of ErpA rescues the growth of *E. coli* producing *cyclo-*SMDIKG induced by 0.2% arabinose, with no effect without arabinose. **D)** Dose-dependent over-expression of ErpA reduces toxicity of *cyclo-*SMDIKG to the host *E. coli* in a cell viability assay. **E)** *cyclo*-SMDIKG binds to ErpA with a *K*_D_ of 4.3 ± 1.0 μM by MST. All data shown as mean (n = 3) ± SEM.

The alanine scanning results indicated that replacement of G had minimal effect on the antibacterial properties of our lead peptide. We therefore designed and synthesized a variant of this molecule with propargylglycine in this position to enable its further functionalisation for use as a pull-down probe to enable target identification. The resulting cyclic peptide was biotinylated via a click reaction with propargylglycine and biotin­(PEG)_3_-N_3_. (Figure 4A) This cyclic peptide was incubated with *E. coli* lysate, and any resulting peptide/protein complex captured using streptavidin-coated beads. The beads were subsequently washed, and the eluted proteins visualized by SDS-PAGE. We observed a difference between pull-down and control (biotin only) with a band present only in the pull-down at around 12 kDa (Figure 4B). To identify this protein, we submitted the pull-down samples for analysis by mass spectrometry-based proteomics. Eight proteins with the expected mass of 12 kDa were identified as potential targets of *cyclo*-SMDIKG; YeeX, MazF, YidB, RaiA, YcgL, YjbR, ErpA, and YeaO.

One potential antibiotic resistance mechanism in bacteria is overexpression of the target protein.^26^ When the target protein is upregulated, an increase in antibiotic concentration is needed to produce the same physiological effect, and the same concentration of the antibiotic should subsequently produce a less pronounced physiological effect. To shed light on the target of *cyclo-*SMDIKG, we assessed the ability of each of the proteins identified from the pull-downs to confer resistance to *cyclo-*SMDIKG when overexpressed. We generated plasmids encoding each of the above proteins under control of a tac promoter to allow IPTG-induced expression of the target protein. Each plasmid was transformed into *E. coli* containing a SICLOPPS plasmid encoding *cyclo*-SMDIKG and the growth of the resulting strain was monitored for 20 h in the presence of 0 and 0.2% arabinose to induce production of *cyclo*-SMDIKG, and 0, 10 μM, 50 μM, 100 μM, and 500 μM IPTG to induce expression of the potential target protein (Figure S8). Only ErpA overexpression conferred an IPTG-dependent growth advantage to the *E. coli* host, with no effect from IPTG observed without arabinose (Figure 4C). To further confirm the ability of ErpA overexpression to suppress *cyclo*-SMDIKG activity, we used a commercial bacterial cell viability assay based on quantification of a luminescent signal produced by luciferase in an ATP-dependent manner,^27^ and observed an IPTG-dependent growth advantage in the presence of 0.2% arabinose (Figure 4D), supporting our findings in the growth assay. ErpA is an A-type carrier protein that binds iron–sulfur clusters and transfers them to apoproteins lacking an iron–sulfur cluster cofactor to drive their maturation to functional holoproteins.^28^ Previous studies have shown that ErpA is essential for both aerobic and anaerobic respiration in *E. coli*.^29^

To assess whether *cyclo*-SMDIKG directly binds ErpA, we produced recombinant ErpA and reconstituted its iron–sulfur cluster to generate the holoenzyme. Cyclic peptide binding was then assessed by MST; *cyclo*-SMDIKG bound holo-ErpA with a dissociation constant of 4.3 ± 1.0 μM (Figure 4E). Together, these data support direct engagement of ErpA by cyclo-SMDIKG and indicate that inhibition of ErpA function underlies the antibacterial activity observed in *E. coli*.

## Discussion

High-throughput screening approaches typically rely on positive selection for hit identification, whereas previous implementations of dropout screening have primarily used gene perturbation technologies (e.g., RNAi or CRISPR) to identify essential genes in mammalian systems.^30,31^ Here, we present a complementary strategy that combines a genetically encoded cyclic peptide library with a ligand-based dropout screen to enable the discovery of antimicrobial molecules directly in living cells.

This approach exploits the structural properties of cyclic hexapeptides, which are conformationally constrained and typically act through contiguous pharmacophores. Although limitations in next-generation sequencing resulted in a large number of apparent dropout sequences, we were able to identify true hits by clustering sequences around shared multi-amino acid motifs (this framework can also be used to identify true hits in enrichment-based screens). Tetrapeptide motifs showed the strongest dropped-out signal, with the MDIK motif emerging as a clear hit. A focused SICLOPPS library containing this motif further validated its activity, and the inability to clone several variants was indicative of their biological activity. Consistent with this, multiple members of the *cyclo-*SMDIKX family exhibited antibacterial activity as synthetic peptides. We selected *cyclo-*SMDIKG as a representative member for further study. This compound inhibited *E. coli* growth in both liquid culture and on solid media, while a scrambled control was inactive. Synthetic samples also showed activity in permeability compromised strains of *E. coli* and *A. baumannii* but not wild type equivalents, consistent with the outer membrane limiting access. Attempts to isolate resistant mutants were dominated by mutations reducing cyclic peptide expression. We therefore employed chemical proteomics and genetic rescue experiments as complementary approaches, which converged on ErpA as the target protein.

Although previously unexplored as an antibacterial target, ErpA is an essential A-type carrier protein responsible for the delivery of iron–sulfur clusters to multiple client proteins involved in respiration and metabolism.^32^ Iron–sulfur cluster transfer by ErpA has been shown to play a central role in a variety of critical pathways,^29,33–36^ and disruption of ErpA function impairs core cellular processes, consistent with the growth inhibition observed here.^28,37–39^ Our difficulty in isolating stable resistant mutants is consistent with ErpA being encoded by a single essential gene with no known functional paralogue under the conditions tested. To our knowledge, *cyclo*-SMDIKG represents the first reported inhibitor of ErpA, highlighting the ability of this approach to identify and access underexplored antibacterial targets.

The screening approach described here enables the identification of bioactive ligands and their intracellular targets, providing a starting point for antibiotic development. Future work will focus on exploring additional sequences within the MDIK motif family and improving potency and permeability through chemical optimization.

In summary, we demonstrate intracellular negative-selection dropout screening as a scalable strategy for the discovery of antibacterial cyclic peptides and their targets directly in living cells. This platform may be broadly applicable for the discovery of antimicrobial agents against diverse bacterial species.

## Supplementary Material



## Data Availability

The data supporting the findings of this study are available within the paper and its Supporting Information. The Python scripts written for this study are available on the Tavassoli Lab GitHub: https://github.com/TavassoliLab. The raw data underpinning this study are openly available from the University of Southampton data repository at https://pubs.acs.org/doi/10.5258/SOTON/D3972.

## Notes

SICLOPPS mechanism (Figure S1); library coverage (Figure S2); change in motifs upon induction (Figure S3); mutations in pAR-Npu-SMDIKX plasmids (Figure S4); function of *cyclo*-SMDIKG in *E. coli* (Figure S5); activity of *cyclo*-SMDIKG in Gram-negative bacteria (Figure S6); alanine scan of *cyclo*-SMDIKG (Figure S7); potential target overexpression (Figure S8); dipeptide dropout motifs (Table S1); tripeptide dropout motifs (Table S2); material and methods; HPLC and MS data for all cyclic peptides (PDF)
