# Supplementary material for: Identification of Antibacterial Cyclic Peptides with a High-Throughput Cell-Based Dropout Screen
Source: J Am Chem Soc. 2026 Jul 25;148(30):32451–60. doi: 10.1021/jacs.6c09188 (PMC13450390; doi:10.1021/jacs.6c09188)
Supplement: Supplementary file 1 [file ja6c09188_si_001.pdf]

Supplementary Information

Identification of antibacterial cyclic peptides with a high-throughput cell-based dropout screen

Leonie M. Windeln,[1] Lewis W. Mitchell,[1] Agnieszka B. Wisniewska,[1] Alexander McDermott,[1] Scott S. Walker[2], Abbas M. Walji[2], Ali Tavassoli[1, *]

1: School of Chemistry and Chemical Engineering, University of Southampton, Southampton, U.K.

2: Merck & Co. Inc., West Point, PA, U.S.A.

* e-mail: ali1@soton.ac.uk

Supplementary Figures

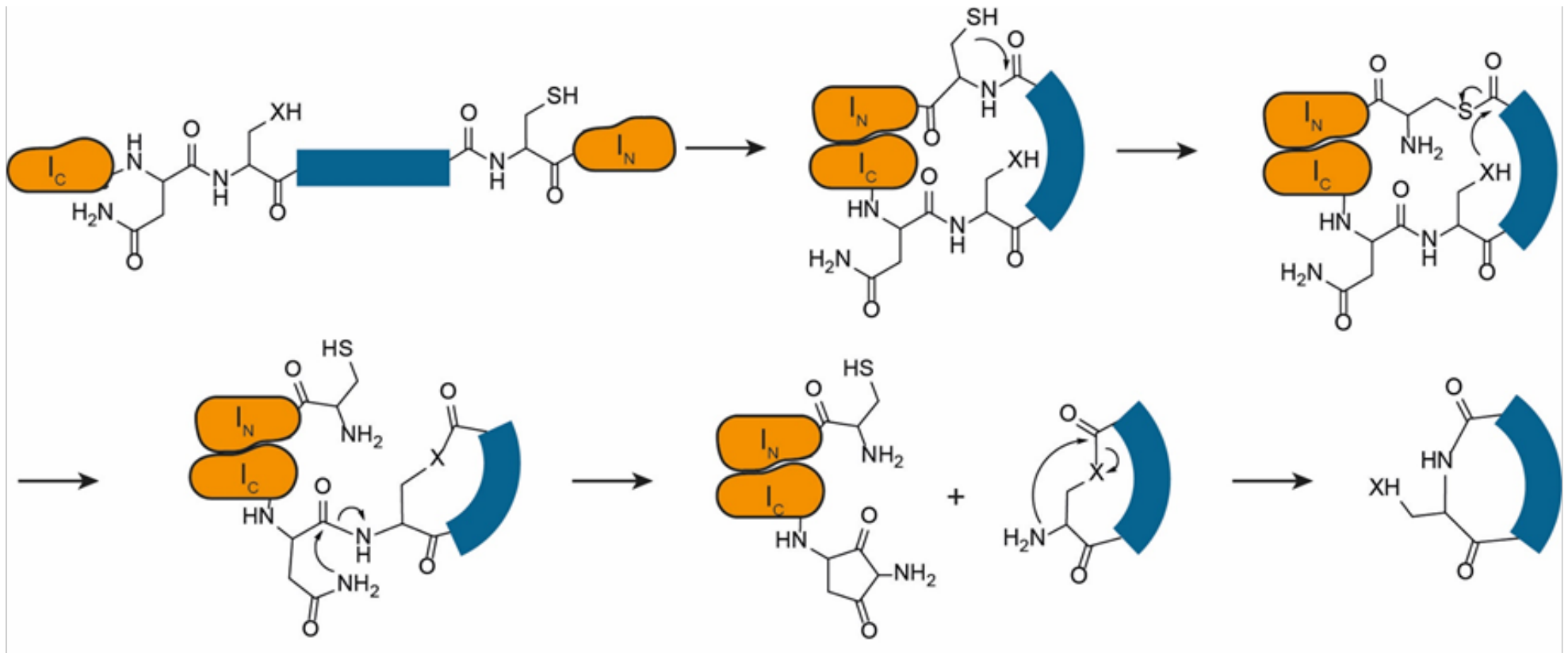

Figure S1. SICLOPPS splicing mechanism. The SICLOPPS intein splices to yield a cyclic peptide (blue) via two nucleophilic attacks and a rearrangement.

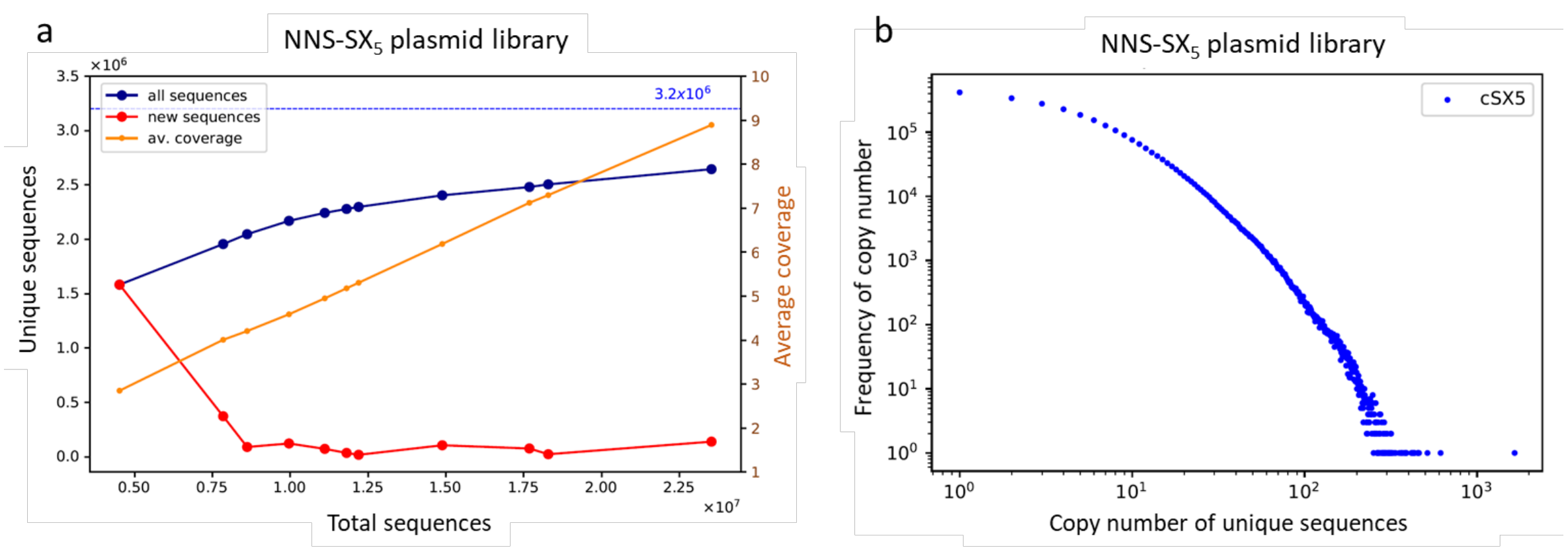

Figure S2. Library coverage. a) Sequence coverage of the plasmid library in relation to the total sequences acquired. Each datapoint represents a different sequencing run. In orange, the calculated average coverage (total sequences/unique sequences). **b)** Coverage plot of all sequences after induction. Each unique sequence is assigned a copy number in the library. The distribution of these copy numbers within the library is then plotted as a frequency. Sequencing of a pre-induction SICLOPPS plasmid library

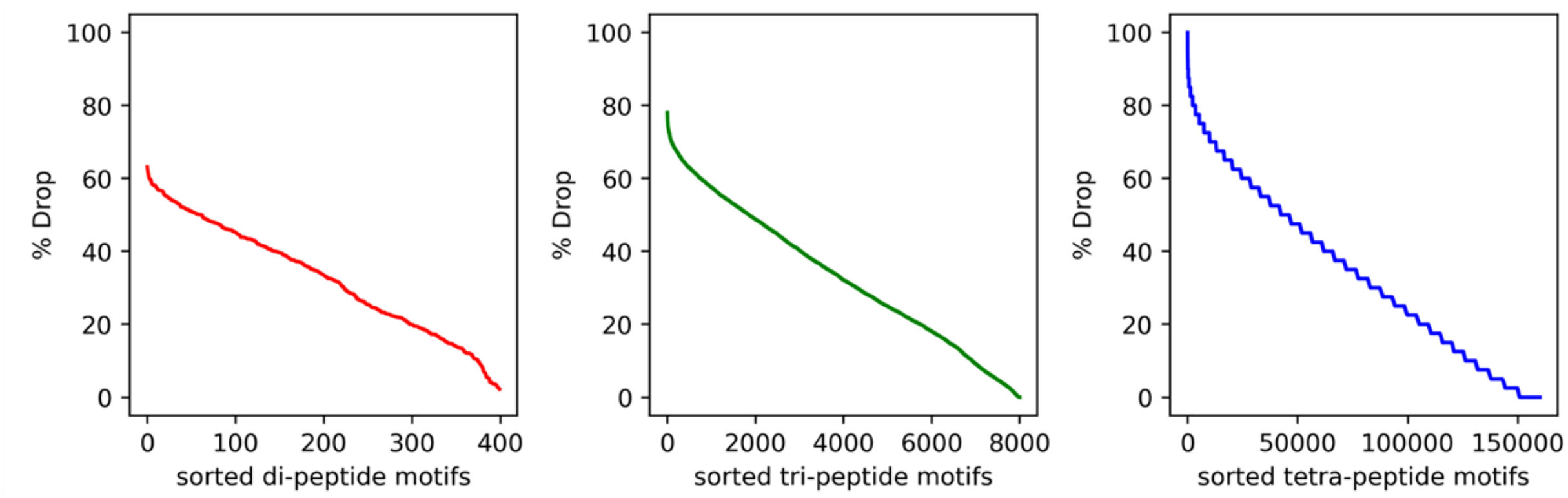

Figure S3. Change in motifs upon induction of a SX_5 library. Decrease (%) of di- (red), tri- (green) and tetra-peptide (blue) sequences within cyclic peptides after induction versus uninduced.

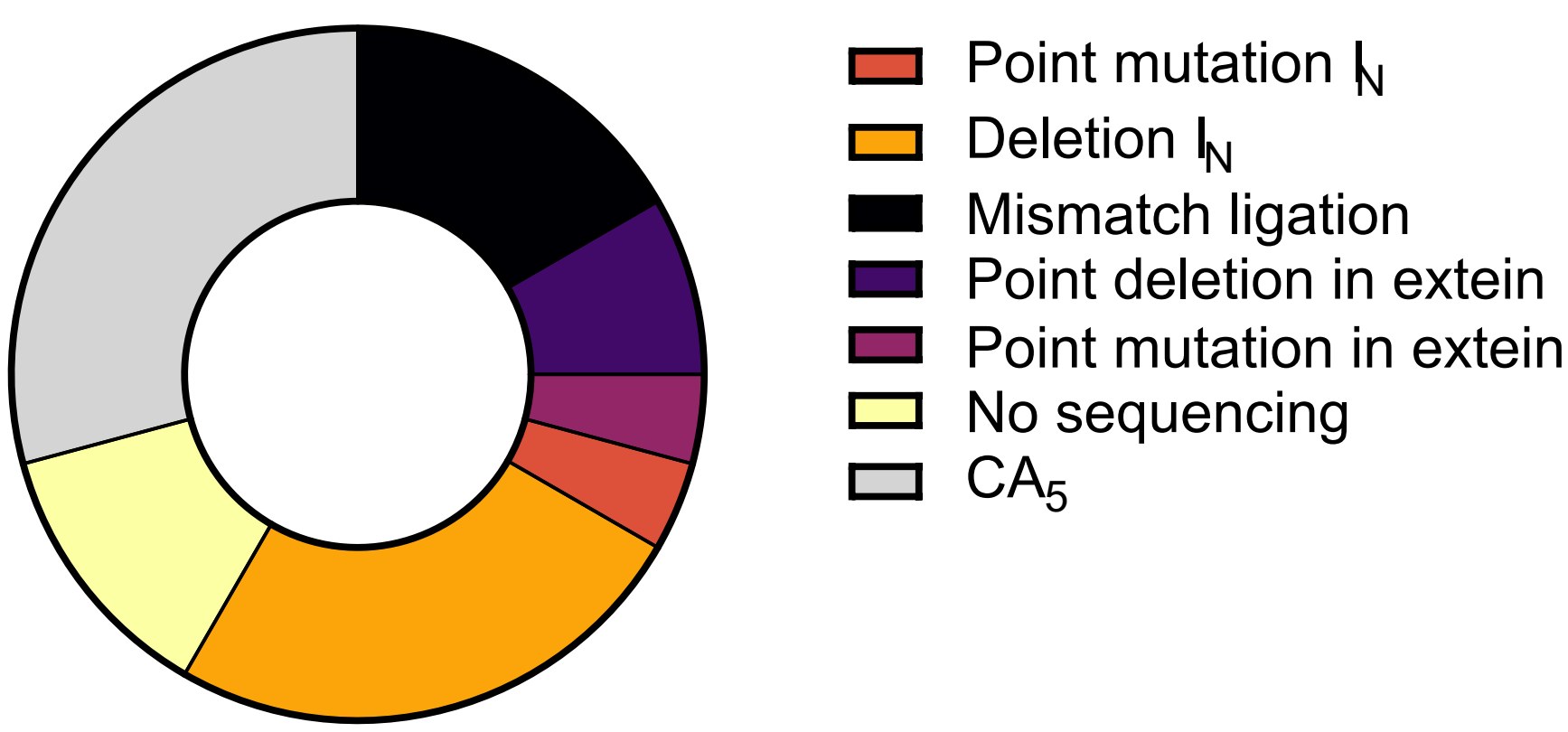

Figure S4. Mutations in pAR-Npu-SMDIKX plasmids. X=A, L, P, Y, D, E, N, H, K, I, T, and Q. Non sequencing samples were samples with less than 200 bp shown in the output file. CA_5 sequences represent vector re-ligation or incomplete digestion.

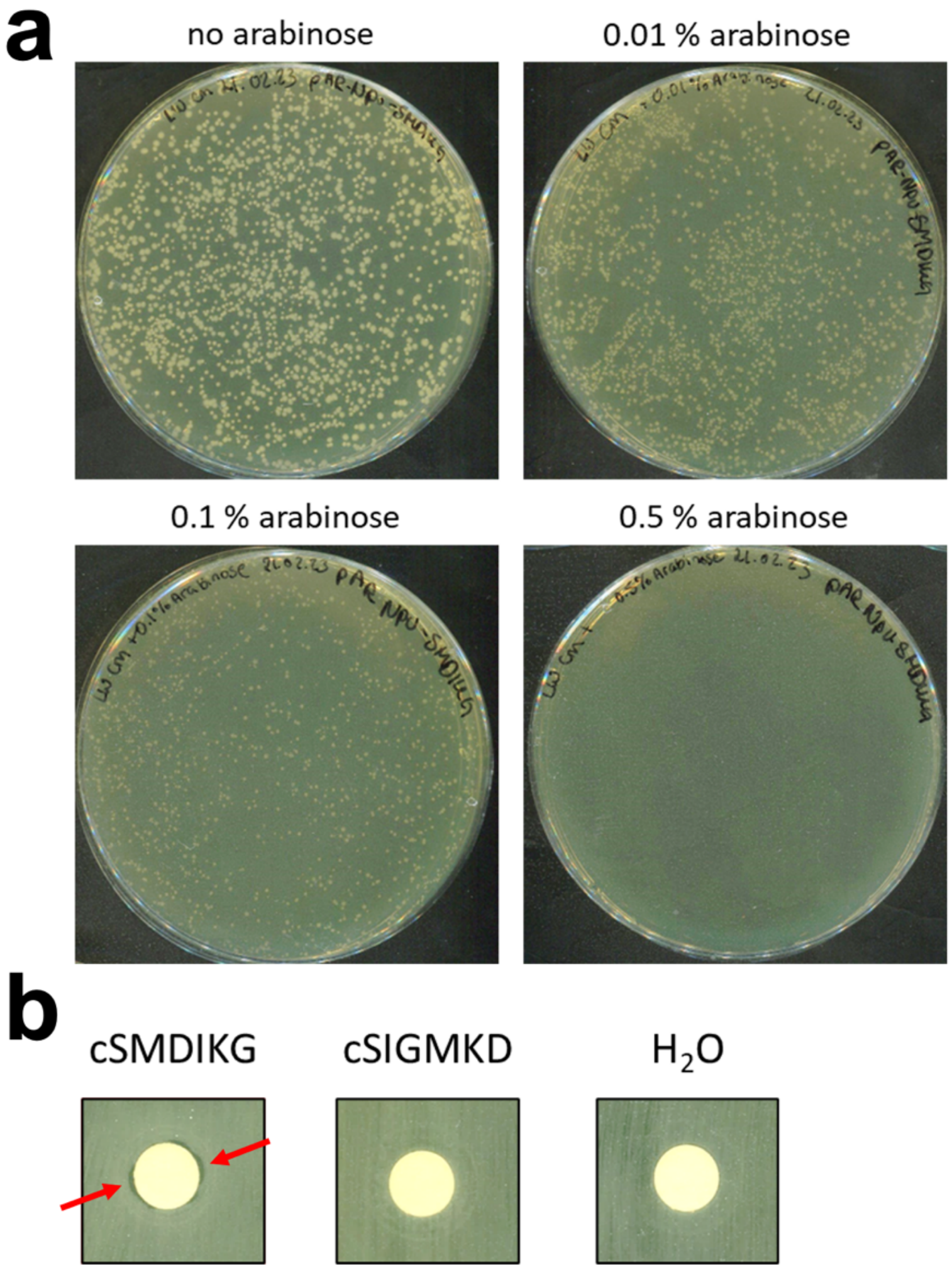

Figure S5. Assessing the function of cyclo-SMDIKG in E. coli. a) Arabinose dependent growth of E. coli DH10β containing a plasmid expressing cyclo-SMDIKG. Fresh chemically competent cells were transformed with pAR-Npu-SMDIKG and 100 µL was plated on LB-agar plates supplemented with chloramphenicol after 1 h recovery. Image taken after 18 h of incubation at 37 °C. **b)** Agar diffusion assay; 4 mm diameter discs were placed on freshly inoculated LB-agar plates, 1 mg compound dissolved in 20 µL dH2O was added per disc; 20 µL dH2O was added on the control disc.

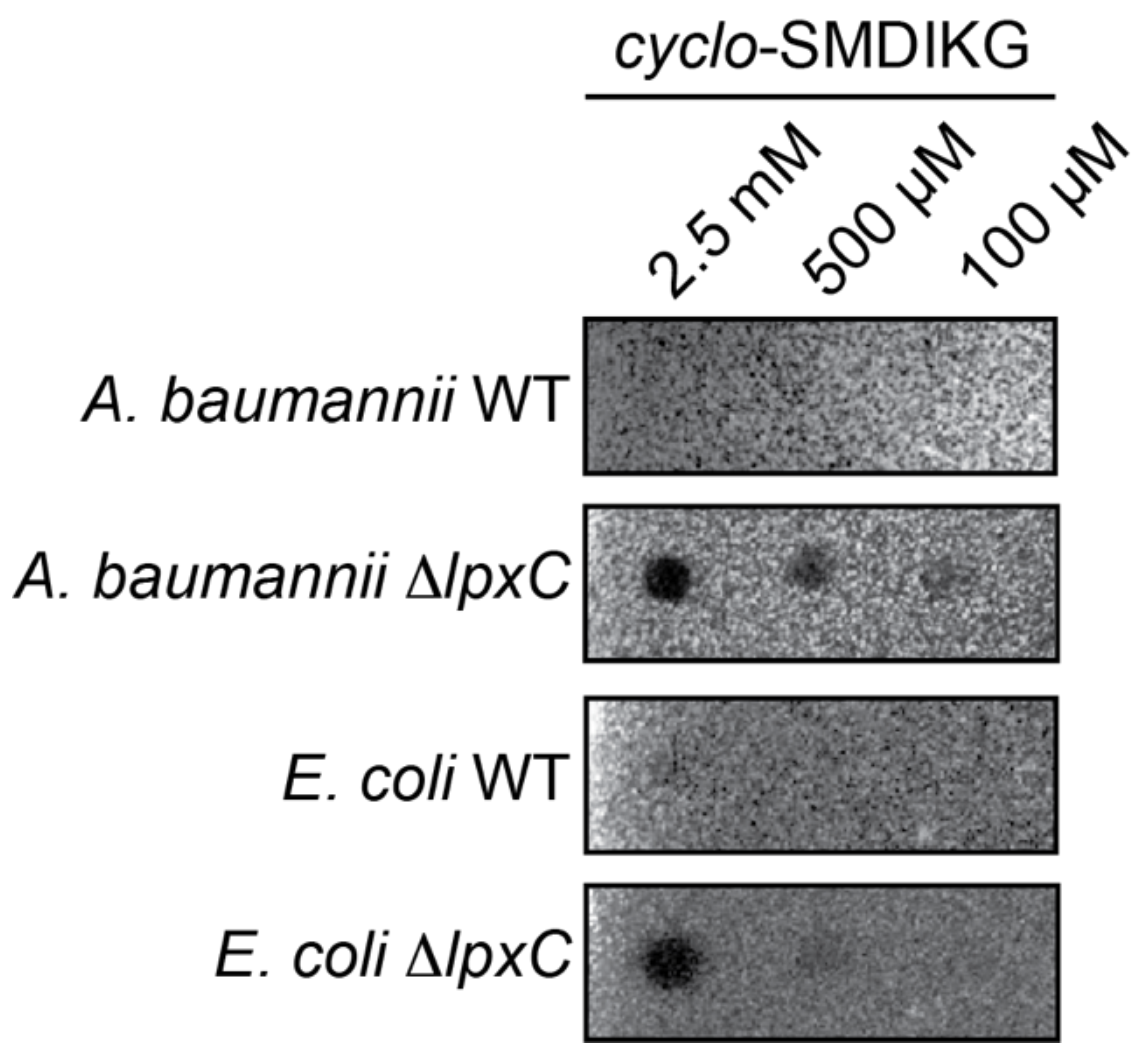

Figure S6. Assessing the activity of *cyclo*-SMDIKG in gram negative bacteria. ZOI assay shows *cyclo*-SMDIKG has no effect on the growth of wild type (WT) E. coli MB4903 and A. baumannii 19606, whereas the growth of the *lpxC* deletion mutant of the same strain is inhibited by this cyclic peptide.

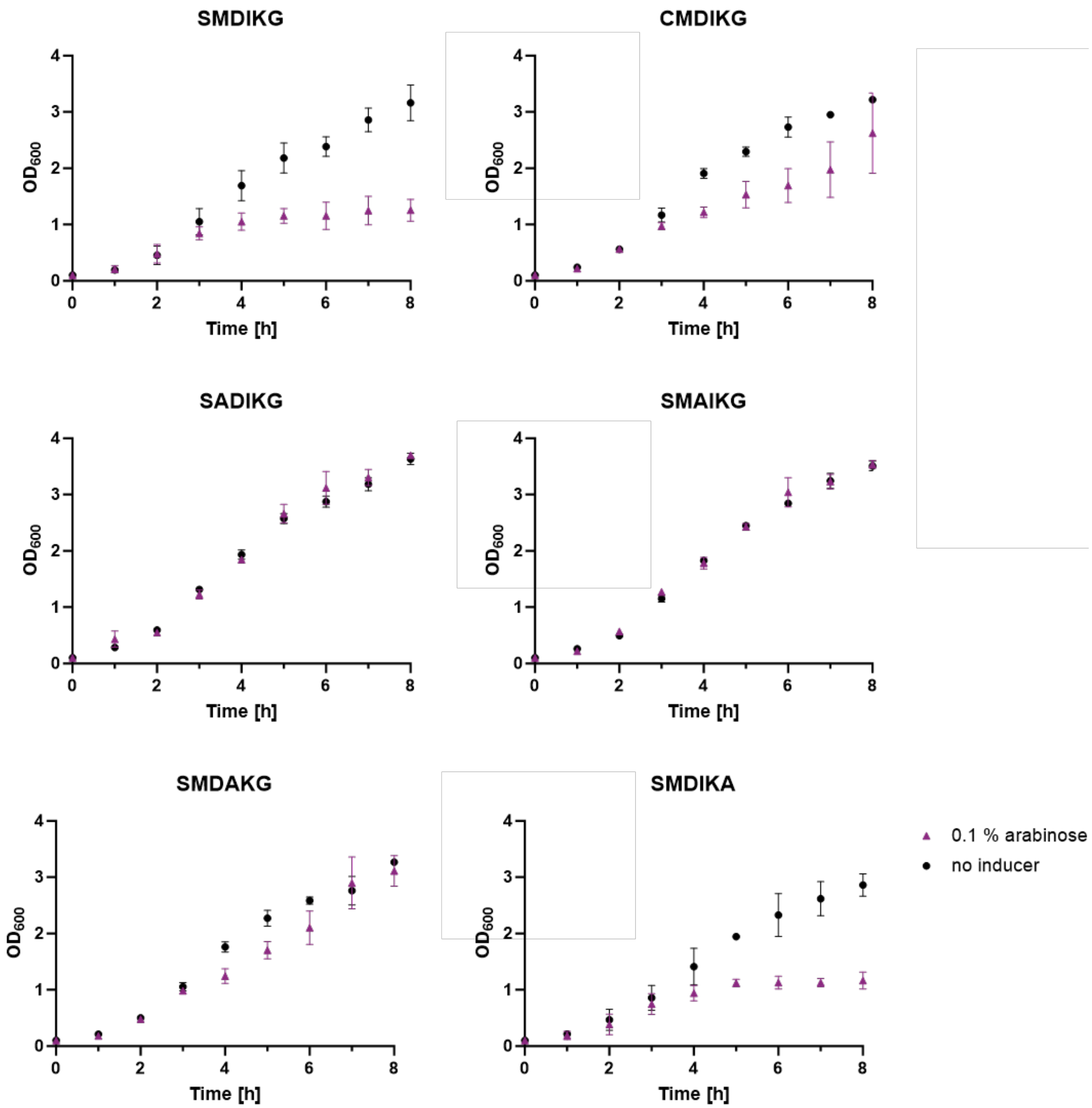

Figure S7. Alanine scan of cyclo-SMDIKG. SICLOPPS plasmids encoding each cyclic peptide were generated and the activity of each on *E. coli* growth was measured when expression was induced by 0.1% arabinose.

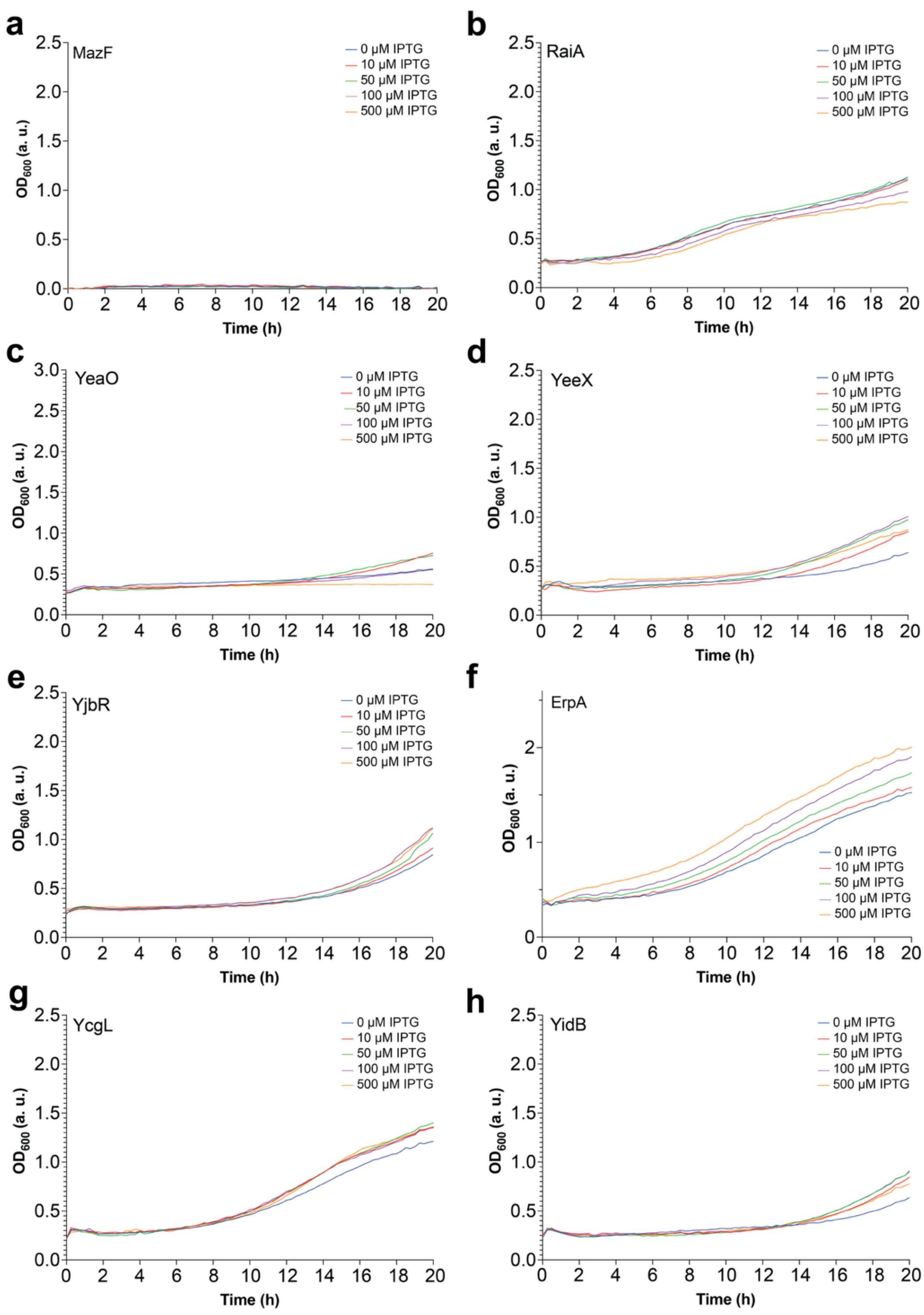

Figure S8. The effect of overexpressing potential targets of *cyclo*-SMDIKG on cell viability. IPTG-driven over-expression of **a)** MazF, **b)** RaiA, **c)** YeaO, **d)** YeeX, **e)** YjbR, **f)** ErpA, **g)** YcgL, **h)** YidB in *E. coli* producing *cyclo*-SMDIKG induced by 0.2 % arabinose. Blank-corrected mean values plotted (n = 3). Error bars omitted for clarity.

Table S1. Dipeptide motif dropout ranking (selected motifs from 400 total). The *cyclo*-SMDIKG motifs reveal a clear dropout gradient: the six MDIK-core dipeptides (blue; MD, DI, IK, DM, ID, KI) all rank in the top 100; the four flanking S- and G-containing dipeptides (orange; SM, MS, KG, GK) rank mid-table and the two G-S motifs (GS, SG) rank near the SAAAAA non-toxic floor. The full dropout dataset is provided in the raw data files.

Motif	Rank	Pre-induction (n)	Post-induction (n)	% Drop
HN	1/400	17,072	12,747	25.3%
HH	2/400	15,768	11,801	25.2%
QN	3/400	17,139	12,908	24.7%
NN	4/400	17,804	13,410	24.7%
NH	5/400	16,248	12,369	23.9%
HK	6/400	17,930	13,652	23.9%
HD	7/400	17,391	13,248	23.8%
DN	8/400	18,626	14,218	23.7%
KN	9/400	18,042	13,828	23.4%
HQ	10/400	16,780	12,867	23.3%
. . .	. . .	. . .	. . .	. . .
KI	44/400	19,201	15,121	21.2%
DI	59/400	20,305	16,210	20.2%
ID	77/400	19,783	16,014	19.1%
DM	74/400	21,405	17,295	19.2%
MD	79/400	20,587	16,673	19.0%
IK	88/400	20,462	16,651	18.6%
. . .	. . .	. . .	. . .	. . .
MS	190/400	147,363	127,039	13.8%
GK	230/400	27,499	24,167	12.1%
SM	234/400	148,708	131,085	11.9%
KG	263/400	28,092	25,023	10.9%
. . .	. . .	. . .	. . .	. . .
SG	330/400	175,150	161,404	7.8%
GS	334/400	177,732	164,088	7.7%
. . .	. . .	. . .	. . .	. . .
SS	357/400	324,905	303,551	6.6%
AA	352/400	29,277	27,273	6.8%
AS	345/400	171,240	158,932	7.2%
SA	294/400	165,409	149,649	9.5%

Table S2. Tripeptide motif dropout ranking (from 1,600 total). MDIK constituent tripeptides MDI and DIK, and single-alanine motifs present at the bottom of the rankings. At the tripeptide level, the MDIK constituents MDI and DIK rank mid-distribution and not distinguishable as a coherent pharmacophore signal, while SAAAAA-derived tripeptides (AAA, SAA, AAS etc.) are absent. The full dropout dataset is provided in the raw data files

Motif	Rank	Pre-induction (n)	Post-induction (n)	% Drop
HNH	1/1,600	445	264	40.7%
QYN	2/1,600	580	352	39.3%
KNN	3/1,600	500	314	37.2%
HIN	4/1,600	568	358	37.0%
KNH	5/1,600	449	284	36.7%
HHD	6/1,600	472	299	36.7%
QKH	7/1,600	464	294	36.6%
IDN	8/1,600	590	375	36.4%
HNC	9/1,600	599	387	35.4%
ENH	10/1,600	501	327	34.7%
. . .	. . .	. . .	. . .	. . .
DIK	504/1,600	647	474	26.7%
MDI	662/1,600	570	424	25.6%
. . .	. . .	. . .	. . .	. . .
HAH	1,486/1,600	691	551	20.3%
HDA	1,553/1,600	756	611	19.2%
YAQ	1,465/1,600	785	625	20.4%
NAH	1,468/1,600	746	594	20.4%
AIH	1,394/1,600	744	586	21.2%

Methods

Generate SX_5 Library

A SICLOPPS library was generated as previously described.[1] The focused libraries and individual sequences were cloned the same way as described for the large library.

Drop out screen

Electrocompetent *E. coli* DH5α were transformed with the plasmid library and plated on LB-plates containing 0.002 % arabinose and 35 µg/mL chloramphenicol. The surviving cells were collected after 18 h and their plasmid DNA was extracted. The entire library was then subjected to NGS as described below.

NGS

Next generation sequencing was performed on an Illumina MiSeq instrument and sample prep was carried out as previously described.[2] To obtain enough sequencing depth, multiple runs of sequencing were carried out. All runs were subjected to the same analysis pipeline to extract coding extein sequences. For this, only sequences with the correct inteins and an average of Q30 or higher were taken forward. For the Venn diagram, the difference in unique sequences was calculated. The motif analyser tool was implemented using NumPy.

Liquid culture screen

To investigate the arabinose dependent growth reduction of cells harboring pAR-Npu plasmids encoding for individual cyclic peptides, main cultures of 10 mL LB were inoculated with an OD_{600} of 0.1 of overnight culture. Arabinose and chloramphenicol were added as appropriate (arabinose stock solution 10 %) and the cultures were incubated at 37 °C, 250 rpm. The OD600 was measured every hour for the duration of the experiment using a Cary 100 Bio UV-Vis Spectrometer (Agilent). For this, 100 µL culture was diluted in 900 µL LB. All experiments were carried out in triplicate, using different colonies each time to inoculate the overnight culture.

Solid phase peptide synthesis

The synthesis of the *cyclo*-SMDIKX library was outsourced.

cyclo-SMDIKG was synthesized using solid phase peptide synthesis (SPPS) and then cyclized in solution phase. All Fmoc-amino acids were purchased from Fluorochem Ltd. All other reagents were purchased from Merck KGaA unless stated otherwise. 2-Chlorotrityl chloride resin (2-CTC) (1 eq.) was swelled for 15 min with 10 mL Dichloromethane (DCM) in a sinter

funnel via nitrogen flow. The DCM was then removed, and 1 eq. of Fmoc-Glycine dissolved in 10 mL DCM was added together with 3 eq. of DIPEA (N,N-Diisopropylethylamine) and agitated for 2 h. To endcap any unreacted sites on the resin, 1 mL MeOH was added to the reaction and agitated for 15 more minutes. The resulting loaded resin was washed with 3 x DCM, 3 X DMF (Dimethylformamide) and 3x Et2O (Diethyl ether) and stored at -20 °C until use. To determine the resin loading, approximately 10 mg of dry resin was weight into a glass vial and swelled for 15 mins in 800 µL DMF. Then, 200 µL Piperidine was added to cleave the Fmoc protection group from the glycine for 15 mins. 100 µL of the reaction mixture was diluted 1:10 in DMF and the concentration of free Fmoc was measured by UV absorbance at 301 nm using a NanoDrop Spectrophotometer (ThermoFisher Scientific). Loaded 2-CTC -glycine resin was deprotected with 20 % piperidine in DMF for 15 minutes before loading of the first Fmoc-amino acid (3 eq.) with Oxyma Pure (3 eq.) and DIC(N,N'-Diisopropylcarbodiimide) (3 eq.) in 5 mL DMF. The coupling reaction was agitated for 1-2 h and then washed. Coupling was confirmed via Kaiser test, indicating no free amines were present. The newly coupled amino acid was deprotected, and the next amino acids were coupled as before. The sequences were built up C' to N' (for SMDIKG: GKIDMS, for SIGMKD: GISDKM). Finally, the last amino acid was deprotected. The linear peptides were cleaved from the resin in a glass vial using 10 mL of 20 % HFIP (1,1,1,3,3,3-hexafluroisopropanol) in DCM for 2 h. The peptides were then precipitated and washed using dry ice cold Et_2O. Their mass was confirmed via LC-MS before cyclisation.

For cyclisation, the linear peptides were dissolved in DMF (1 mg/mL) and 3 eq. of DIPEA and HATU were added. The reaction was stirred over night at room temperature. Cyclization was confirmed by mass spectrometry, before the excess DMF was removed and the crude cyclic peptides were purified using preparative HPLC (high pressure liquid chromatography). The peptides were subsequently deprotected in a cleavage mix containing 4.75 mL TFA (Trifluoroacetic acid), 0.125 mL dH_2O and 0.125 mL TIPS (Triisopopylsilane) and stirred for 1 h. Deprotected cyclic peptides were precipitated with dry ice cold Et_2O as before. Afterwards, the deprotected cyclic peptides were purified via HPLC. Pure cyclic peptide was then subjected to both analytical HPLC to confirm purity >95 % and high-resolution mass spectrometry to confirm the mass.

For the synthesis of biotinylated SMDIKG, glycine was replaced by propargyl glycine in the SPPS. After cyclisation, a click reaction with biotin-PEG3 azide was carried out. (0.5 eq. CuI, 1.5 eq. sodium ascorbate, 2 eq. DIPEA in dH_2O).

Zoning assay

Cyclic peptide solutions in sterile dH2O were prepared from 100 to 25 mg/mL. LB- or M9 minimal media plates were prepared by drying them for 10 min at 37 °C and pre indicating the intended dropping pattern with a waterproof marker pen. Then, an overnight culture of E. coli DH5α was diluted 1:4 and 150 µL were plated with a spatula onto a round plate and 1 µL of cyclic peptide solution was dropped onto the marked spots. They were then incubated at 37 °C overnight before imaging.

Pull down assay

For the pull-down assay, a 100 mL main culture of E. coli was inoculated with 1 % overnight culture and grown for 4 h at 37 °C, 220 rpm. The cells were then harvested, resuspended in 10-20 mL PBS (phosphate buffered saline, ThermoFisher Scientific tablets pH 7.4) supplemented with protease inhibitor (PierceTM, 1 tablet per 50 mL), and lysed using sonication. The cell debris was then removed by centrifugation at 8000 rpm for 1 h at 4 °C and the lysate was sterile filtered. The protein concentration in the lysate was determined using the PierceTM BCA assay kit (ThermoFisher Scientific) and adjusted to 0.5-2 mg/mL depending on experiment. Cyclic SMDIKG-biotin was dissolved in PBS to a concentration of 1 mM. 90 µL of lysate were mixed with 10 µL of diluted compound (final concentration 1- 100 µM) and incubated on ice for 2 h or overnight. Then, 5 µL of PierceTM magnetic streptavidin beads (ThermoFisher Scientific) were washed twice in 200 µL PBS prior to use. After the final wash, the beads were resuspended in 10 µL PBS and added to the lysate and probe mix. The assay tubes were incubated for 2 h or overnight on ice with occasional agitation. After the incubation, the supernatant containing unbound proteins was removed using a magnetic separator and the beads were washed twice with 100 µL PBS unless otherwise specified. To elute the bound protein, 15 µL 1x SDS buffer were added and then boiled for 5 min at 95 °C before being analysed via SDS PAGE. For mass spectrometry, the washed beads were transferred to a new tube and washed once more with TBS before shipping to Edinburgh proteomics facility.

Analytical data

cyclo-SMDIKG

Analytical HPLC

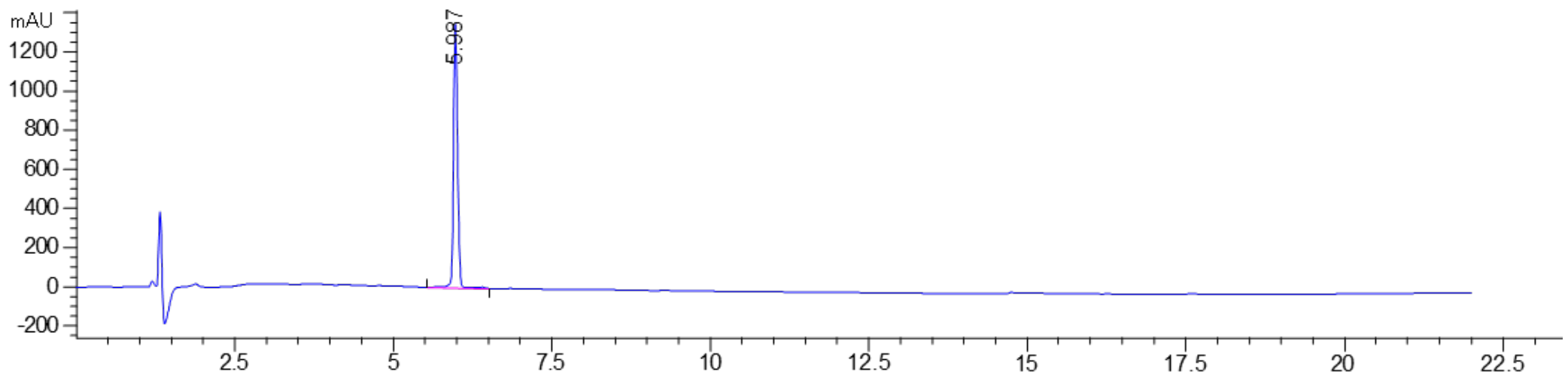

Mass spectrum

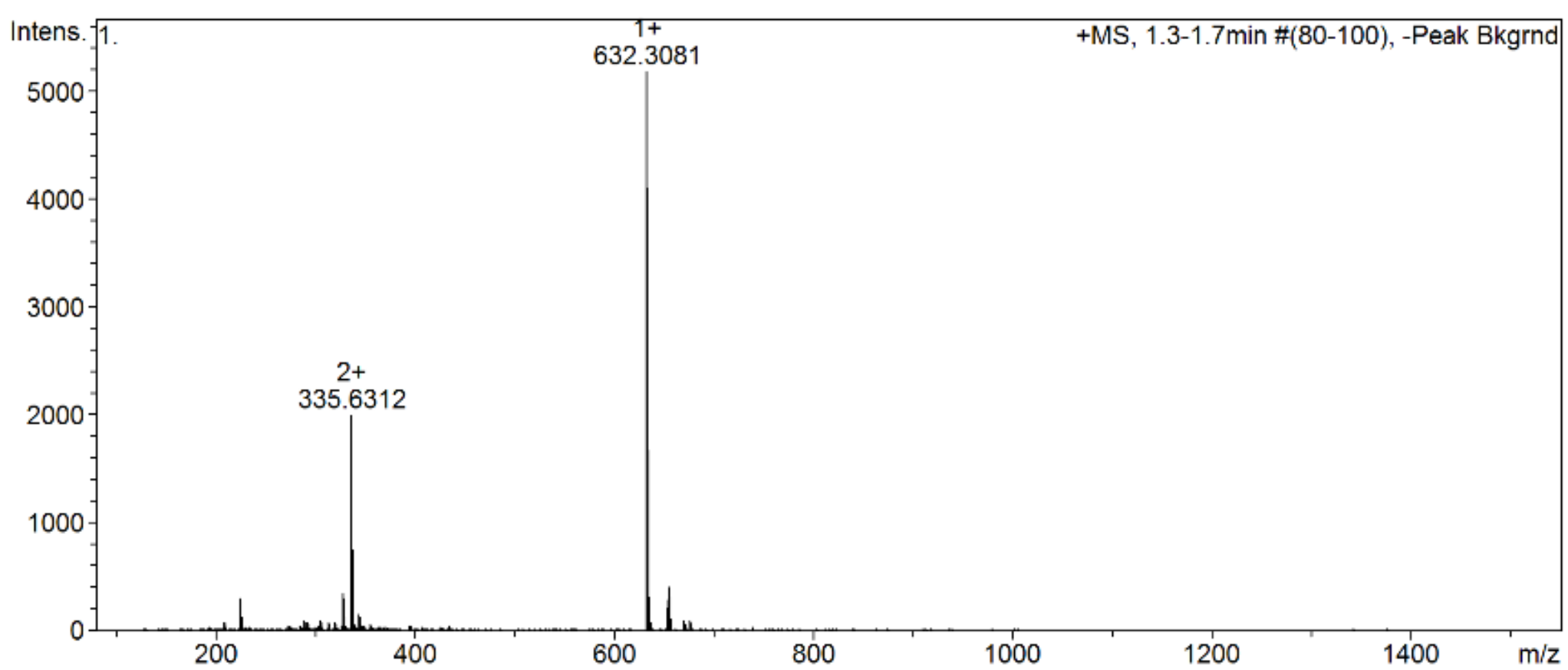

cyclo-SMDIKK

Analytical HPLC

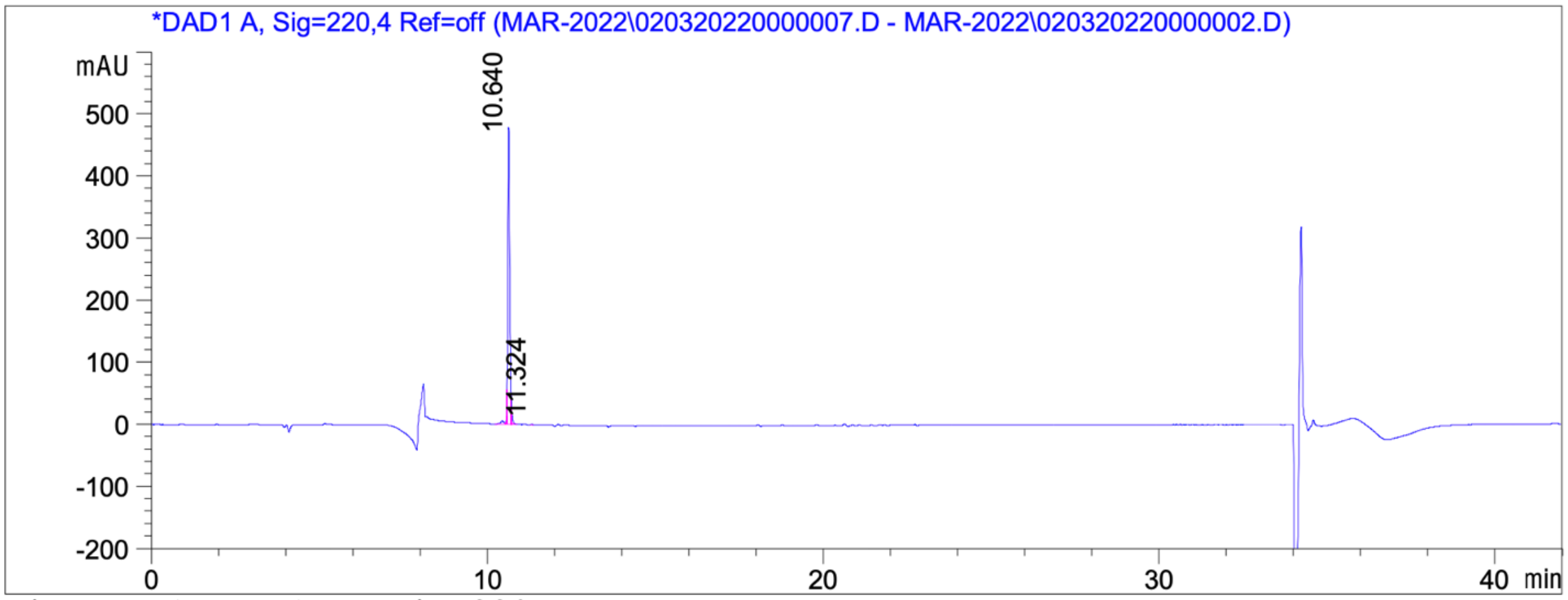

Mass spectrum

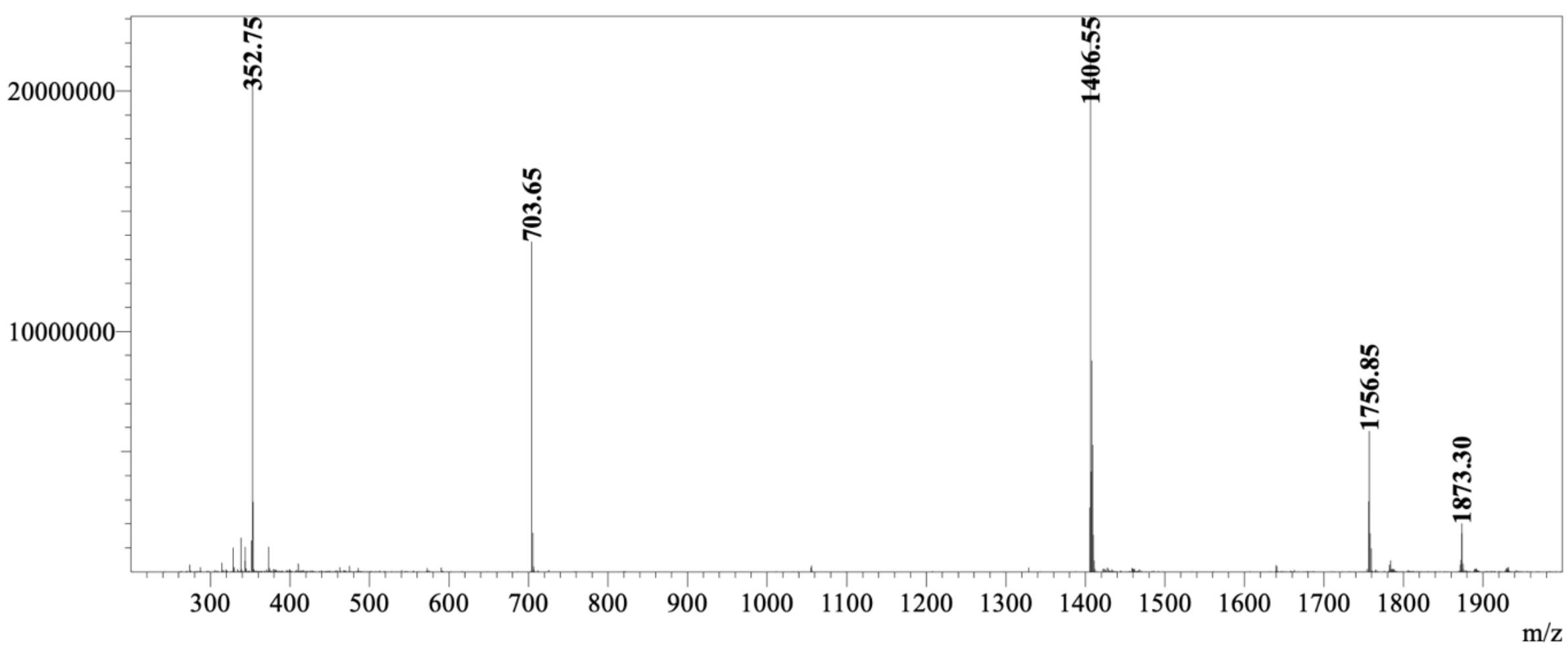

cyclo-SMDIKD

Analytical HPLC

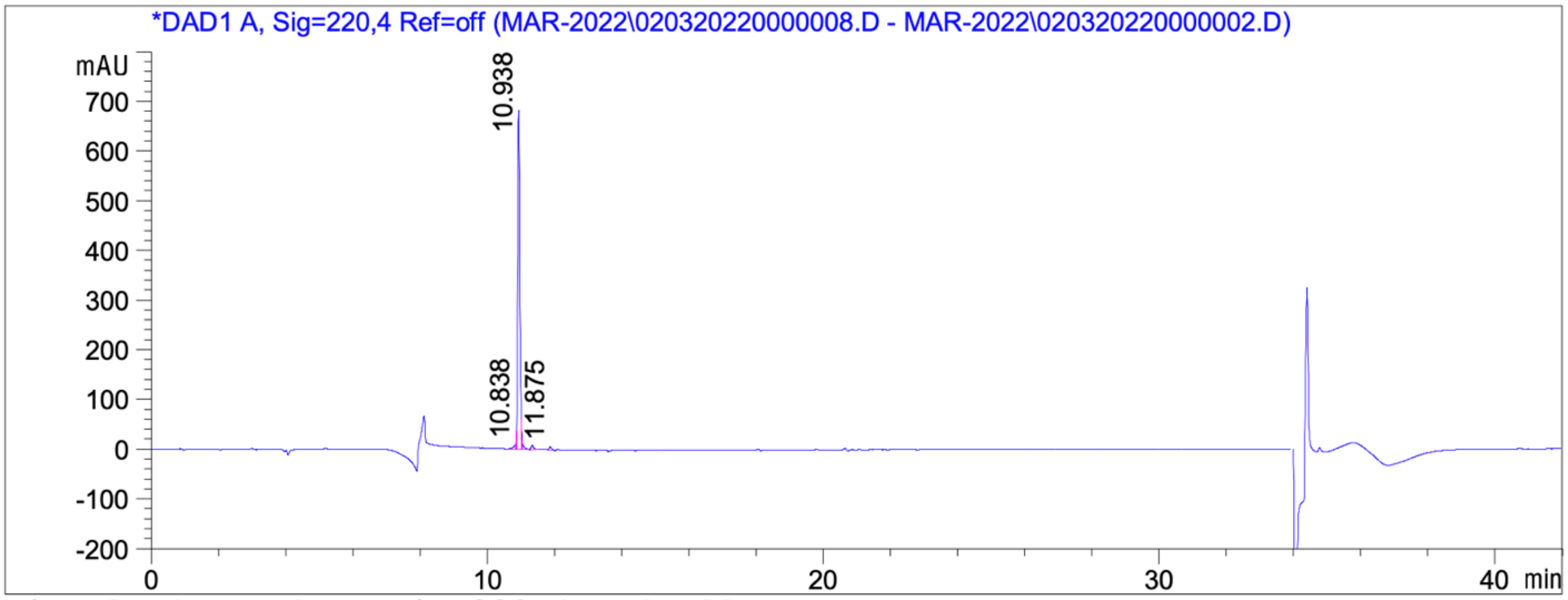

Mass spectrum

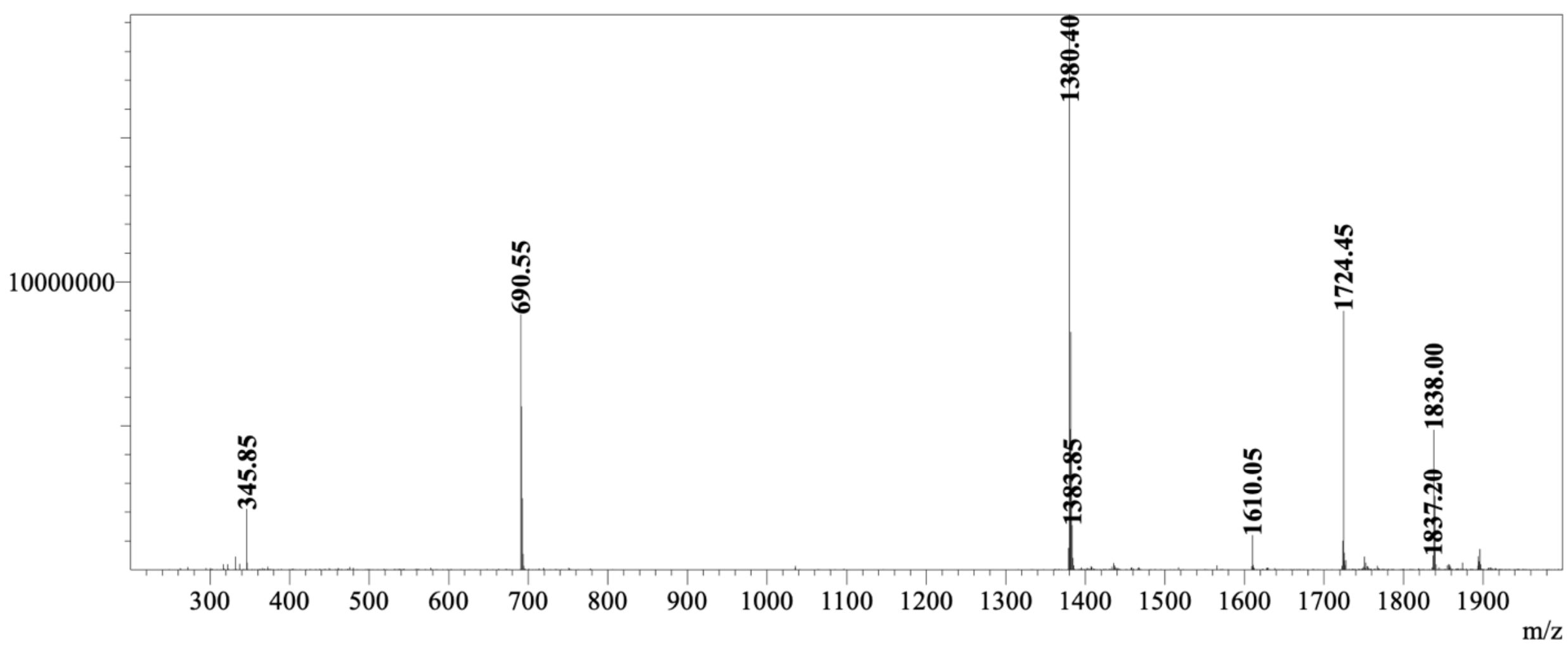

cyclo-SMDIKE

Analytical HPLC

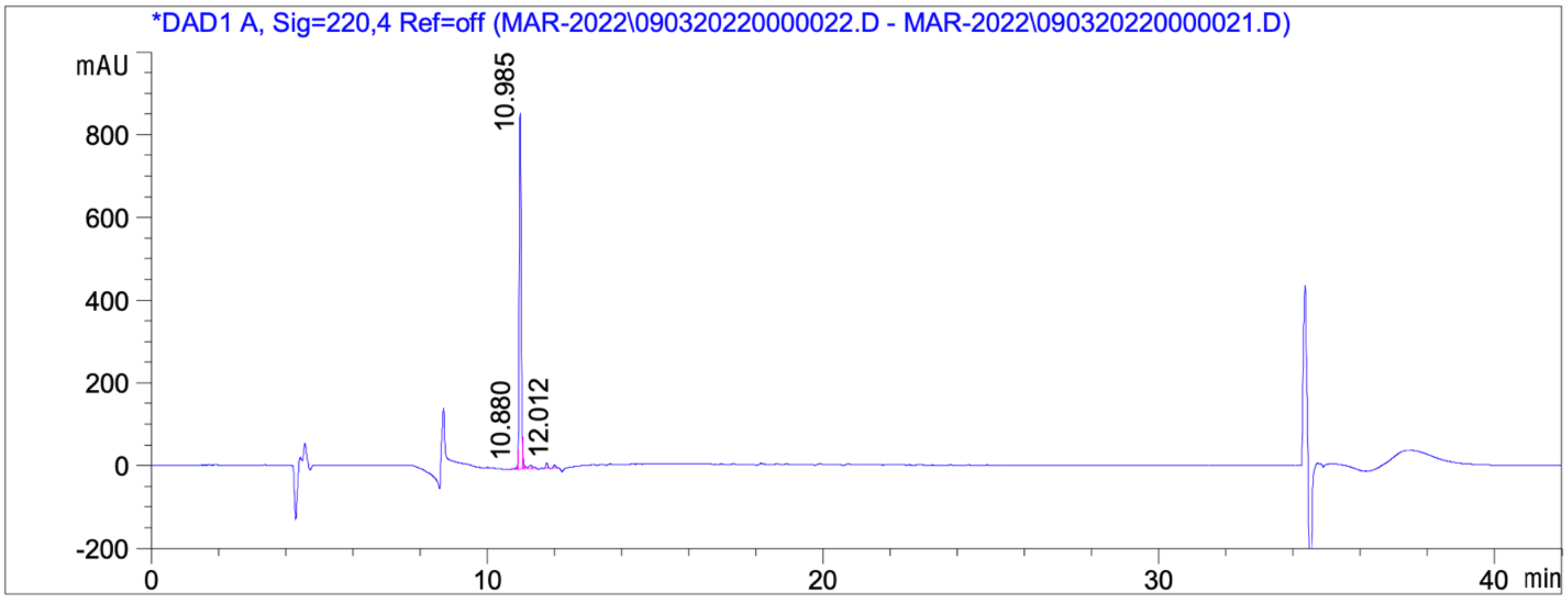

Mass spectrum

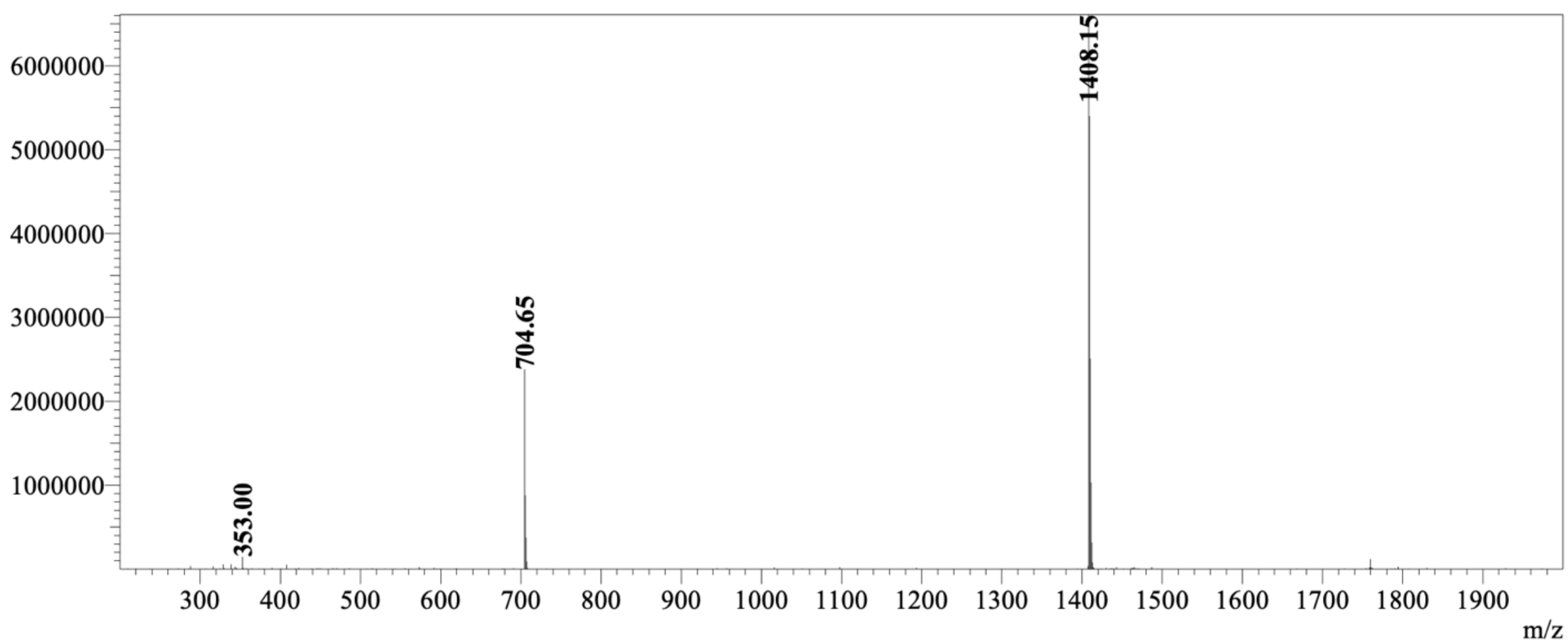

<u>*cyclo*-SMDIKS</u>

Analytical HPLC

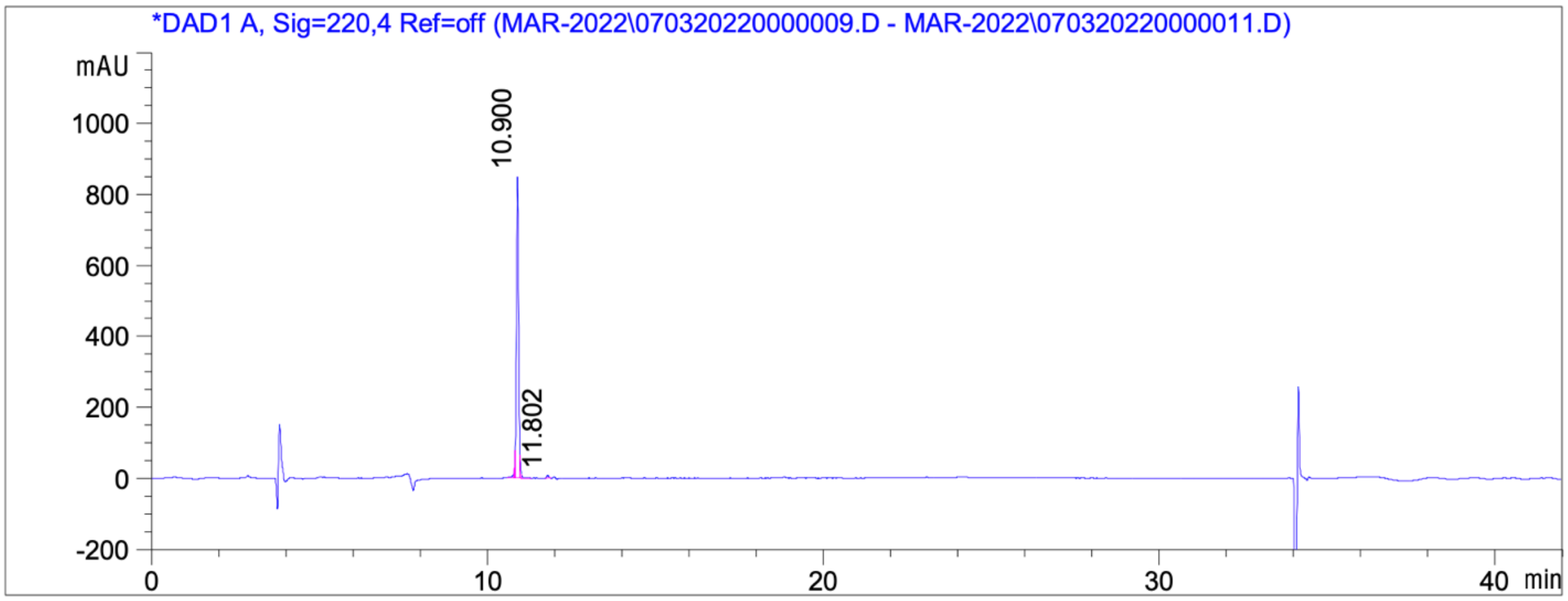

Mass spectrum

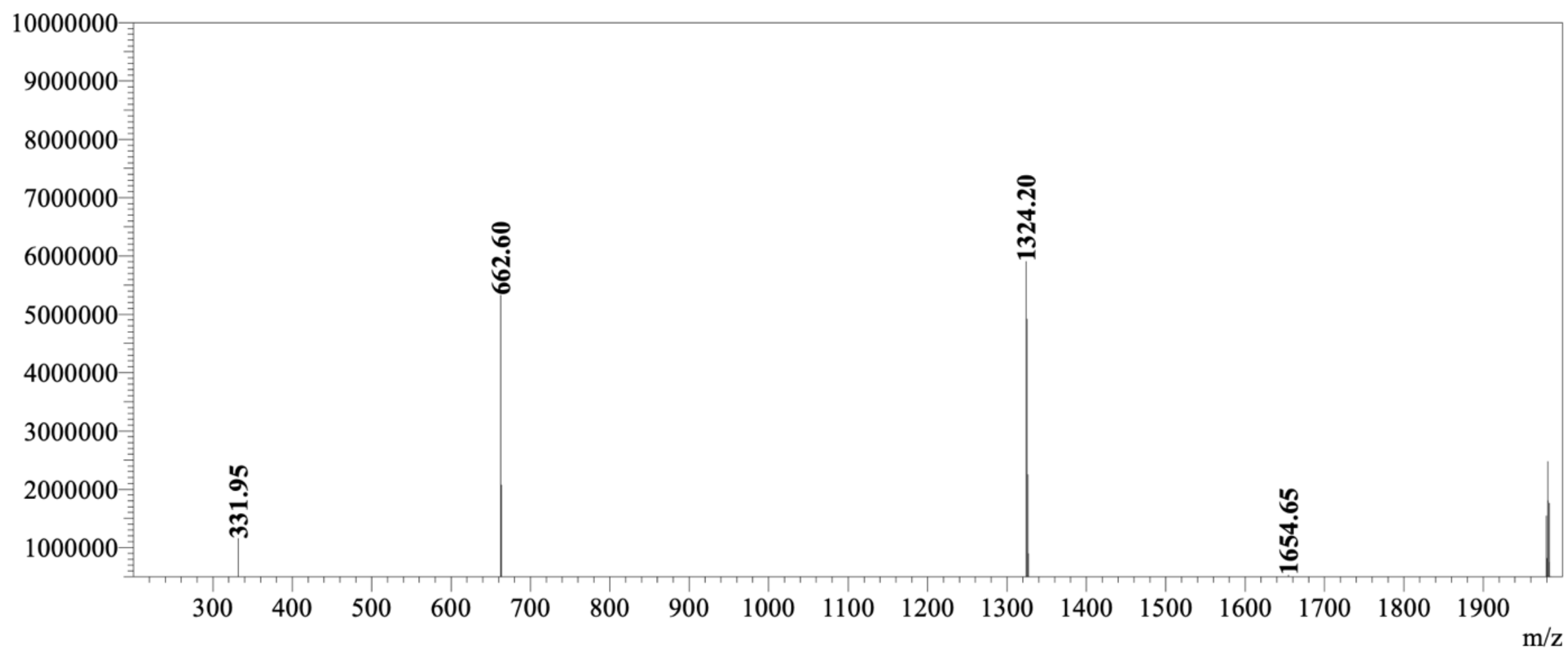

<u>*cyclo*-SMDIKT</u>

Analytical HPLC

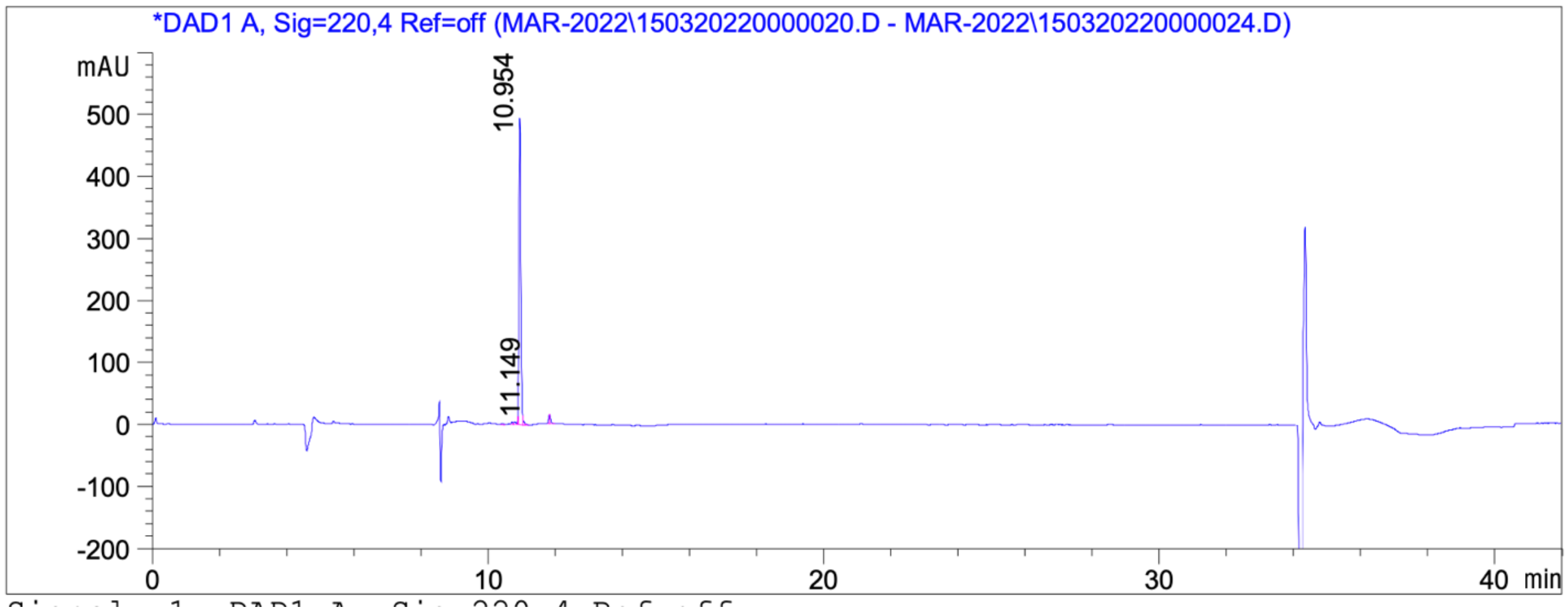

Mass spectrum

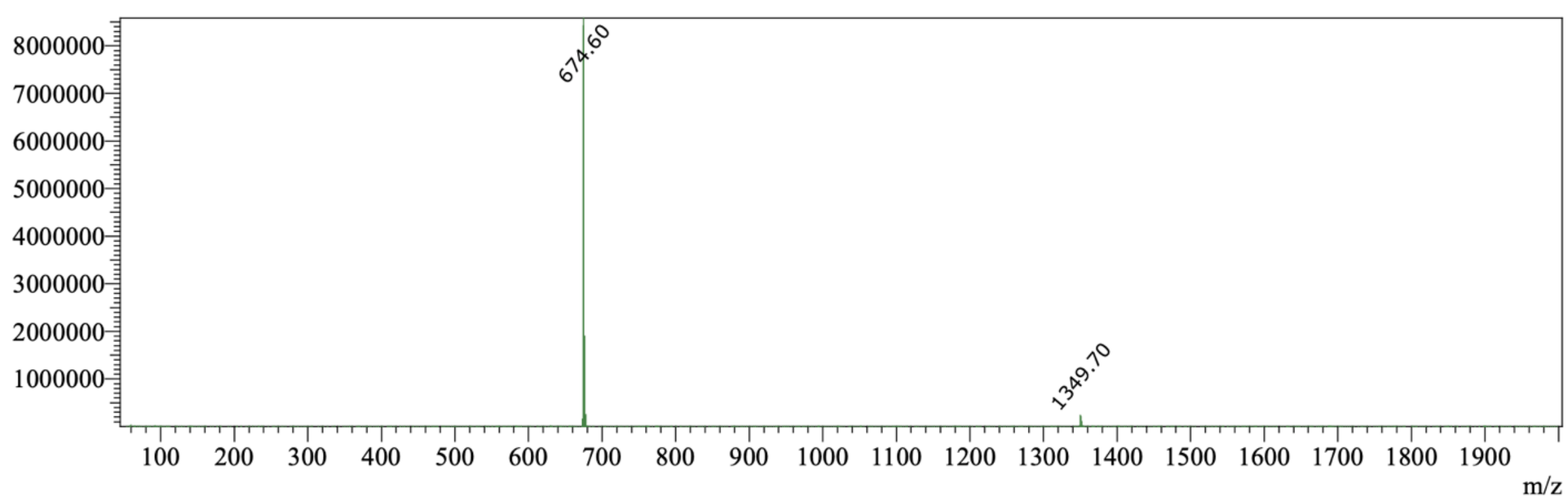

<u>*cyclo*-SMDIKN</u>

Analytical HPLC

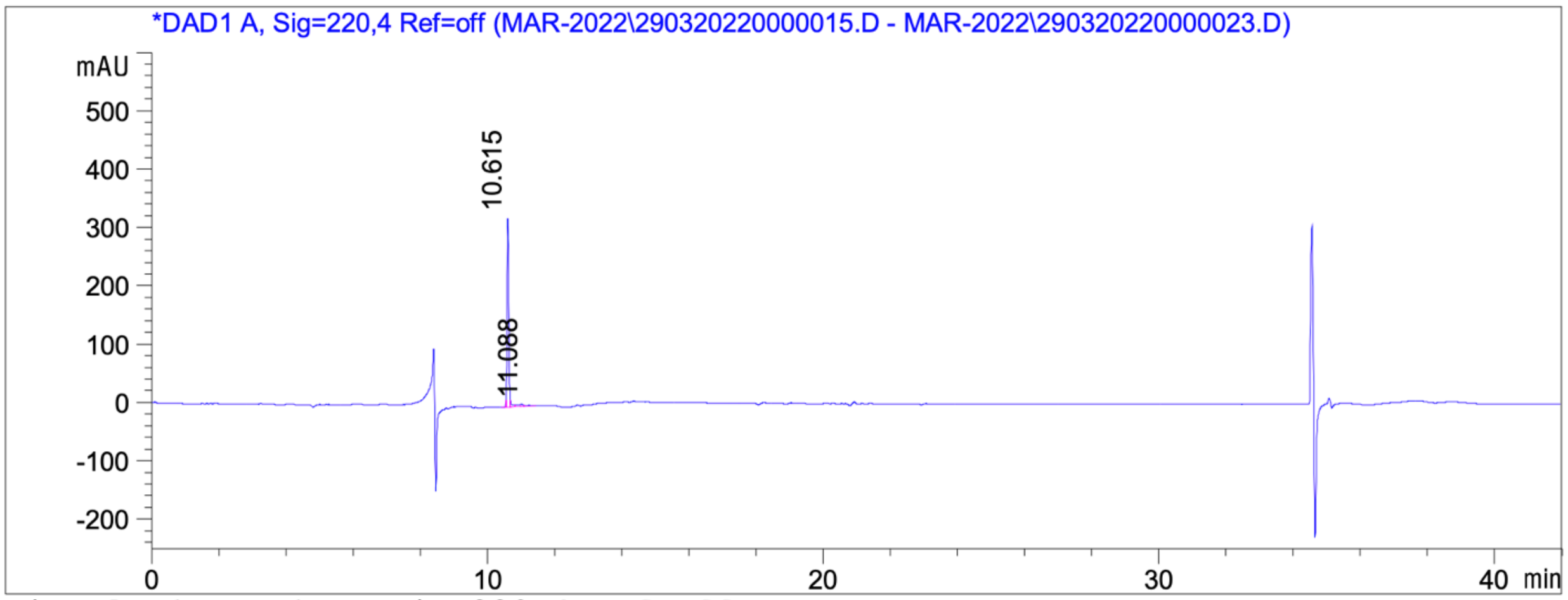

Mass spectrum

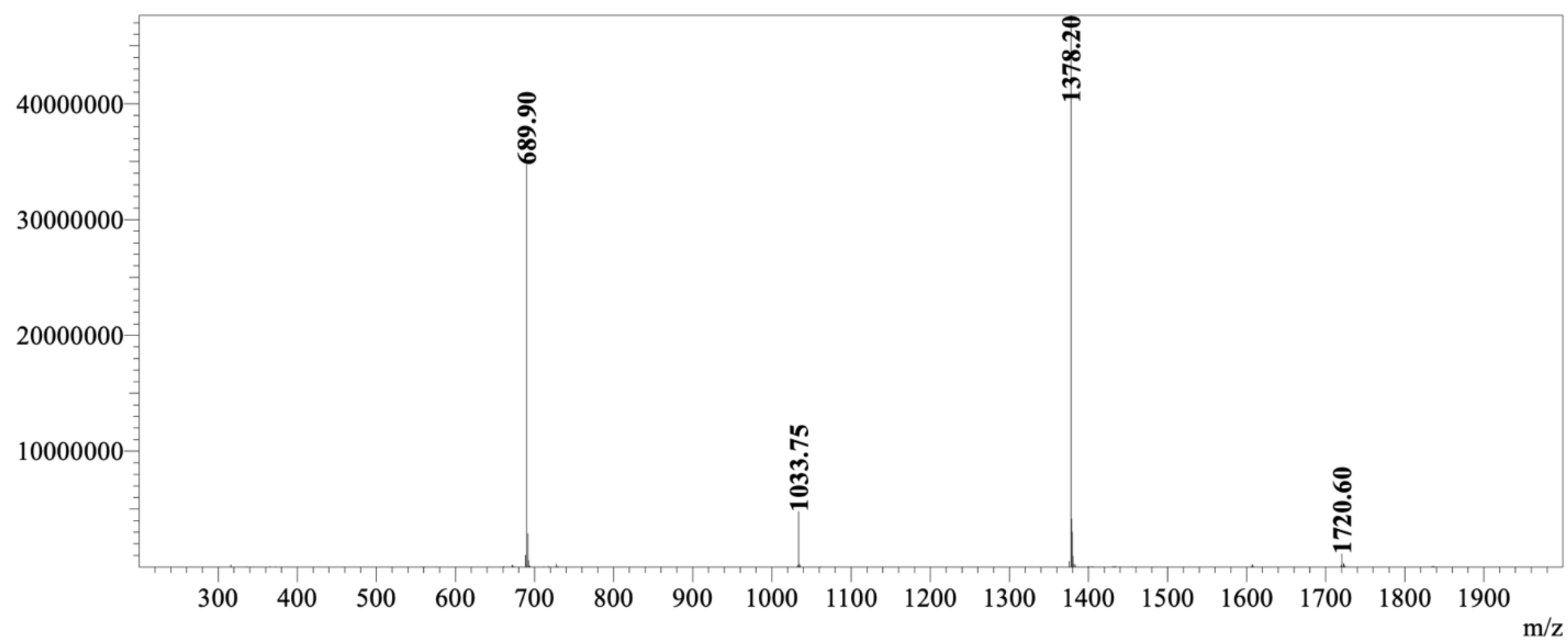

cyclo-SMDIKQ

Analytical HPLC

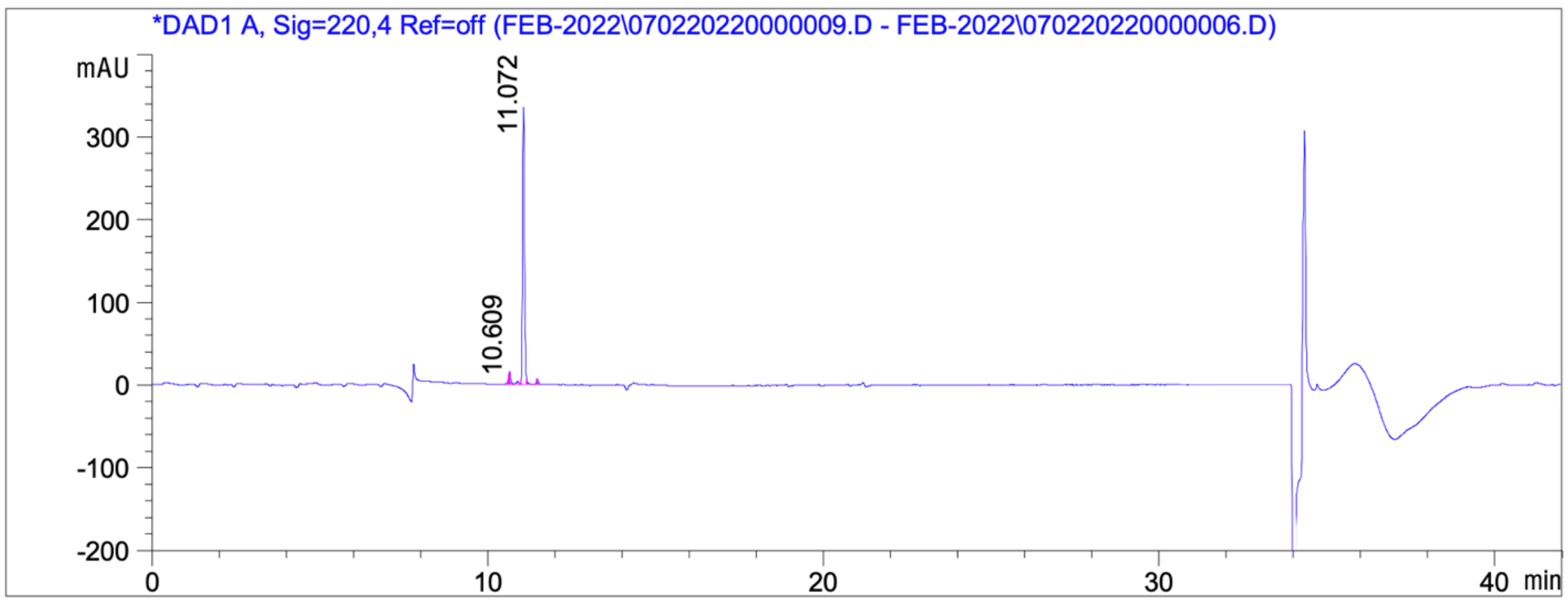

Mass spectrum

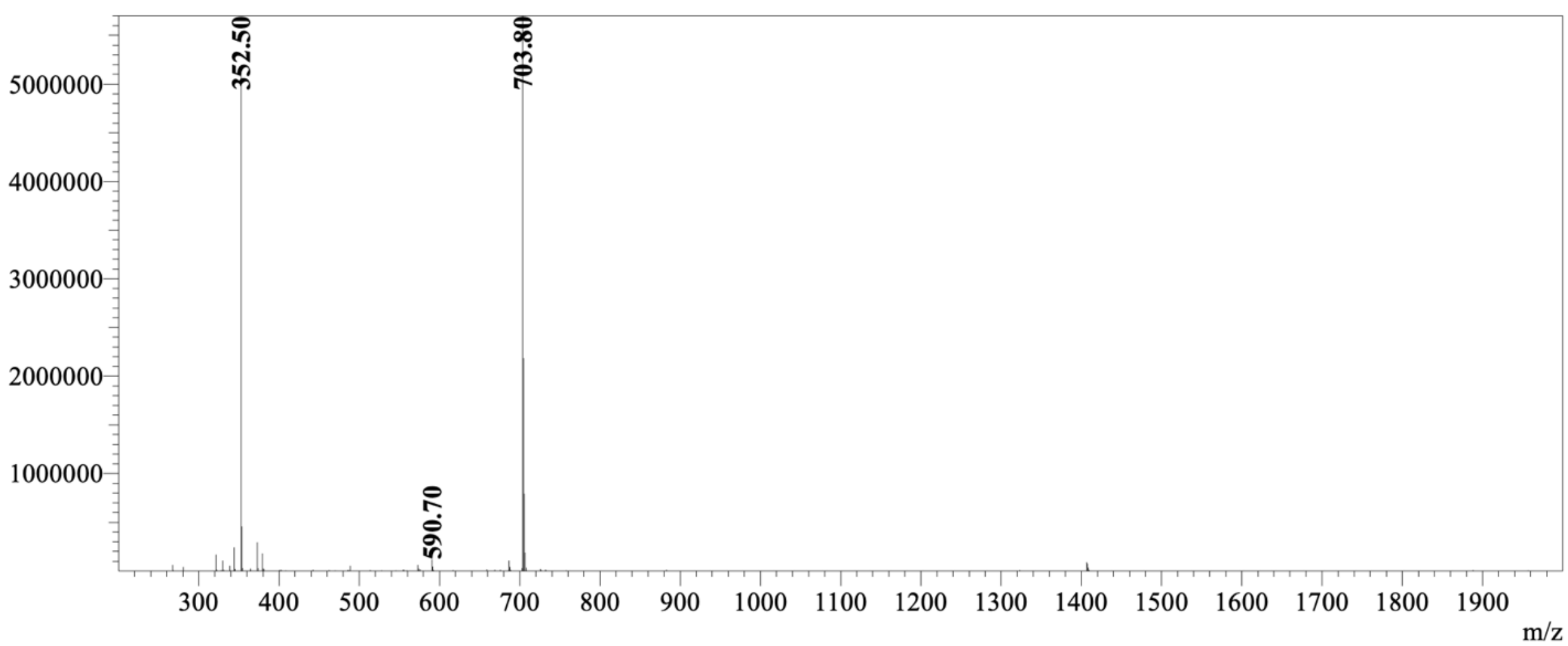

<u>*cyclo*-SMDIKC</u>

Analytical HPLC

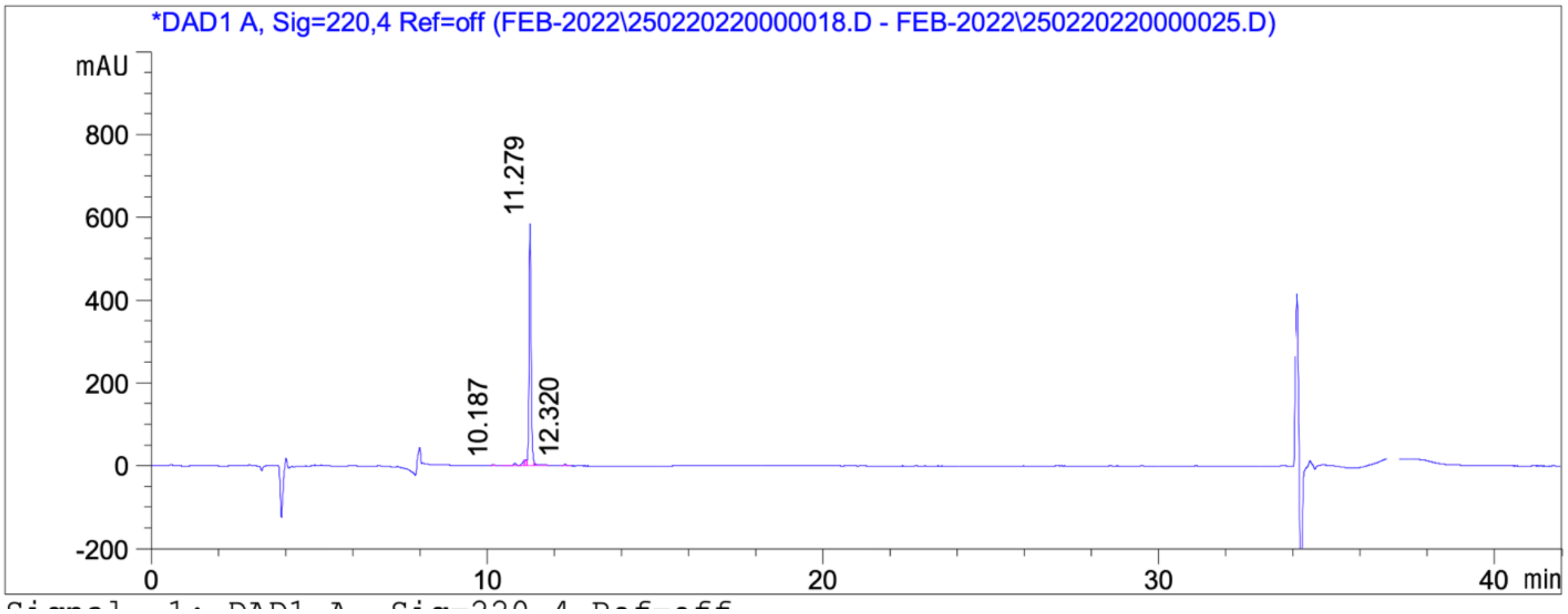

Mass spectrum

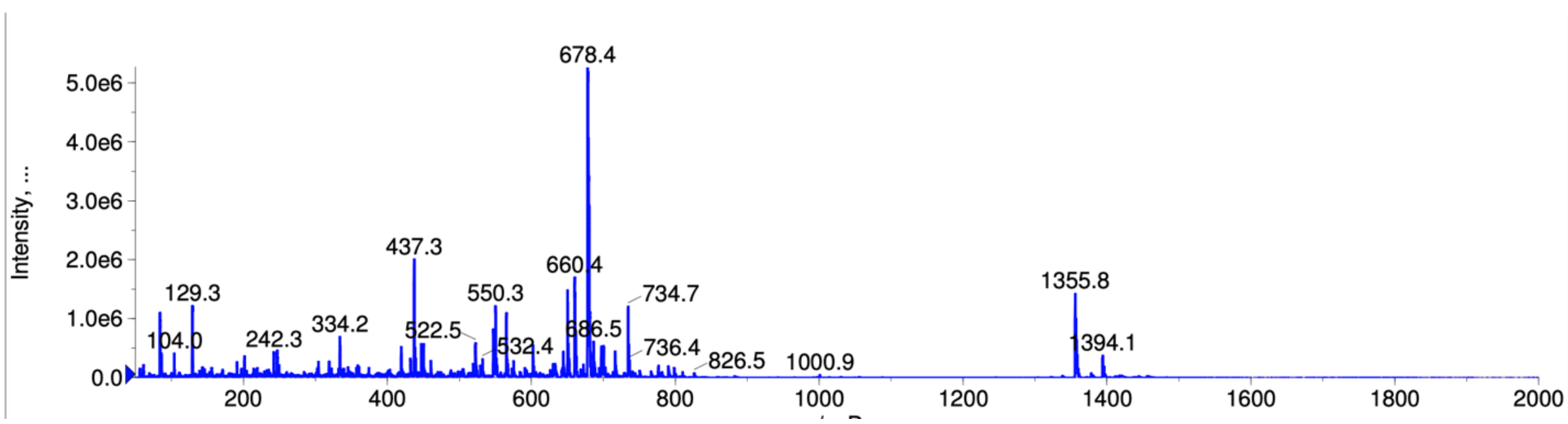

cyclo-SMDIKG

Analytical HPLC

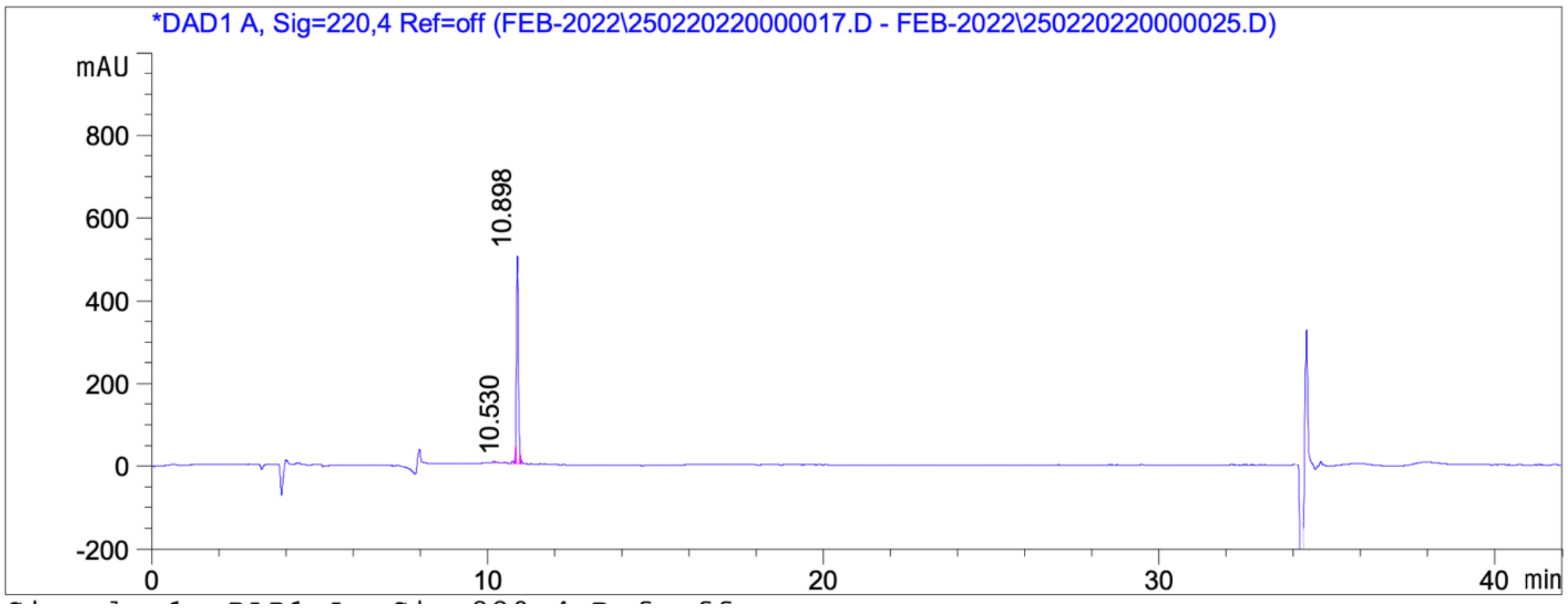

Mass spectrum

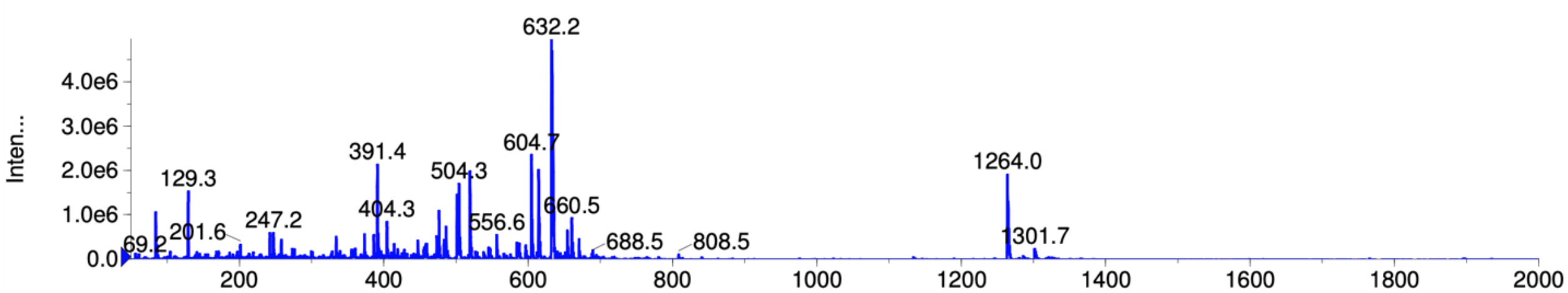

cyclo-SMDIKP

Analytical HPLC

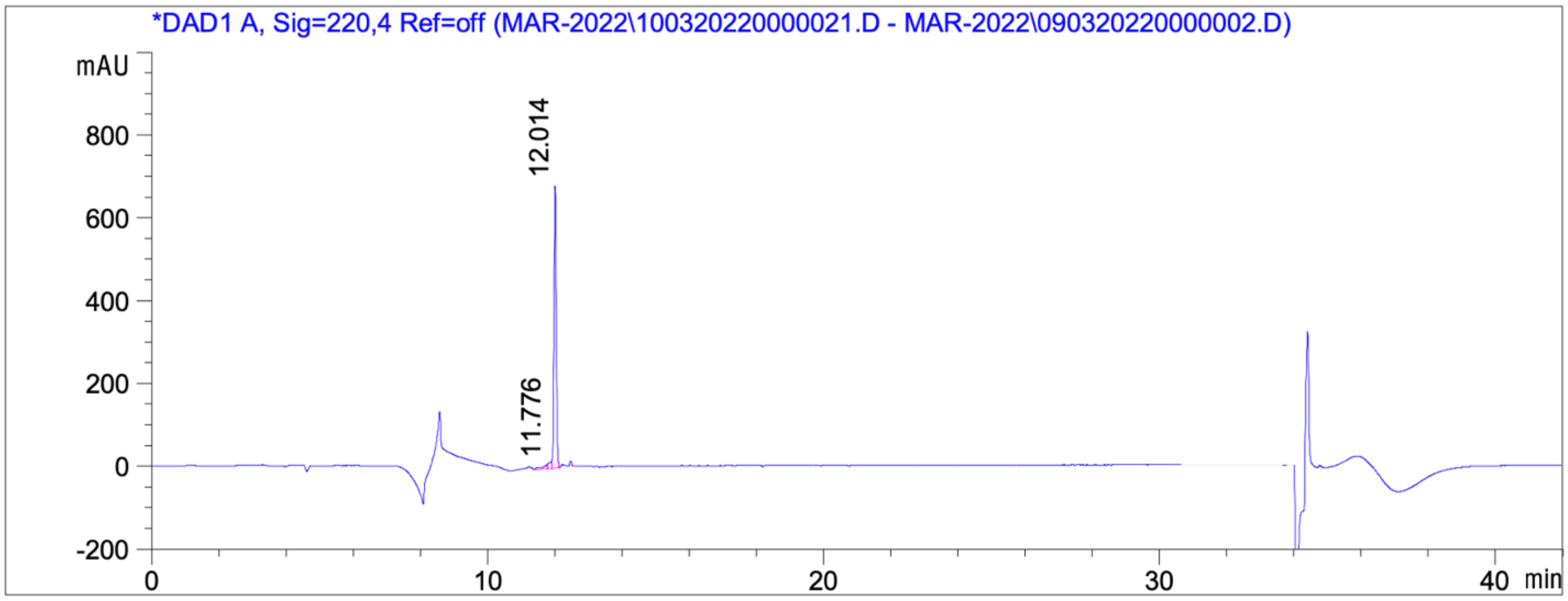

Mass spectrum

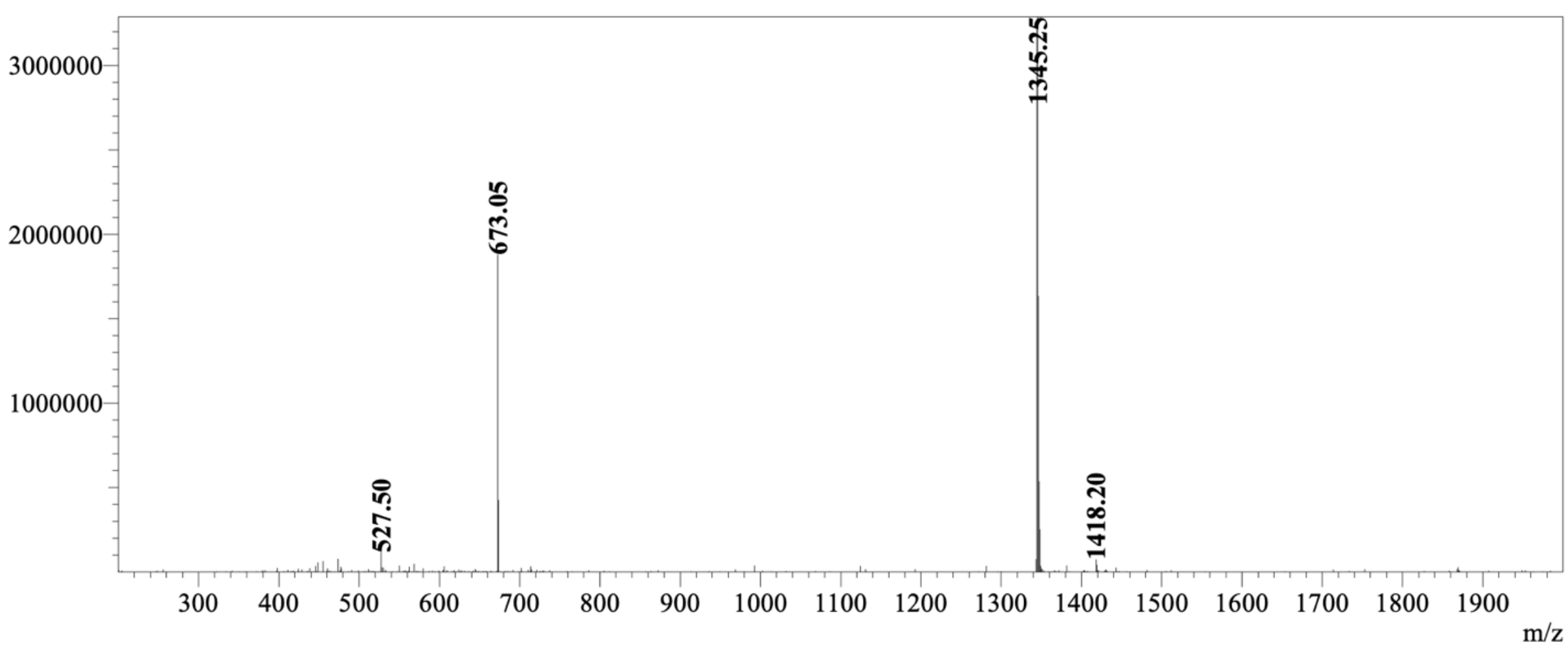

cyclo-SMDIKA

Analytical HPLC

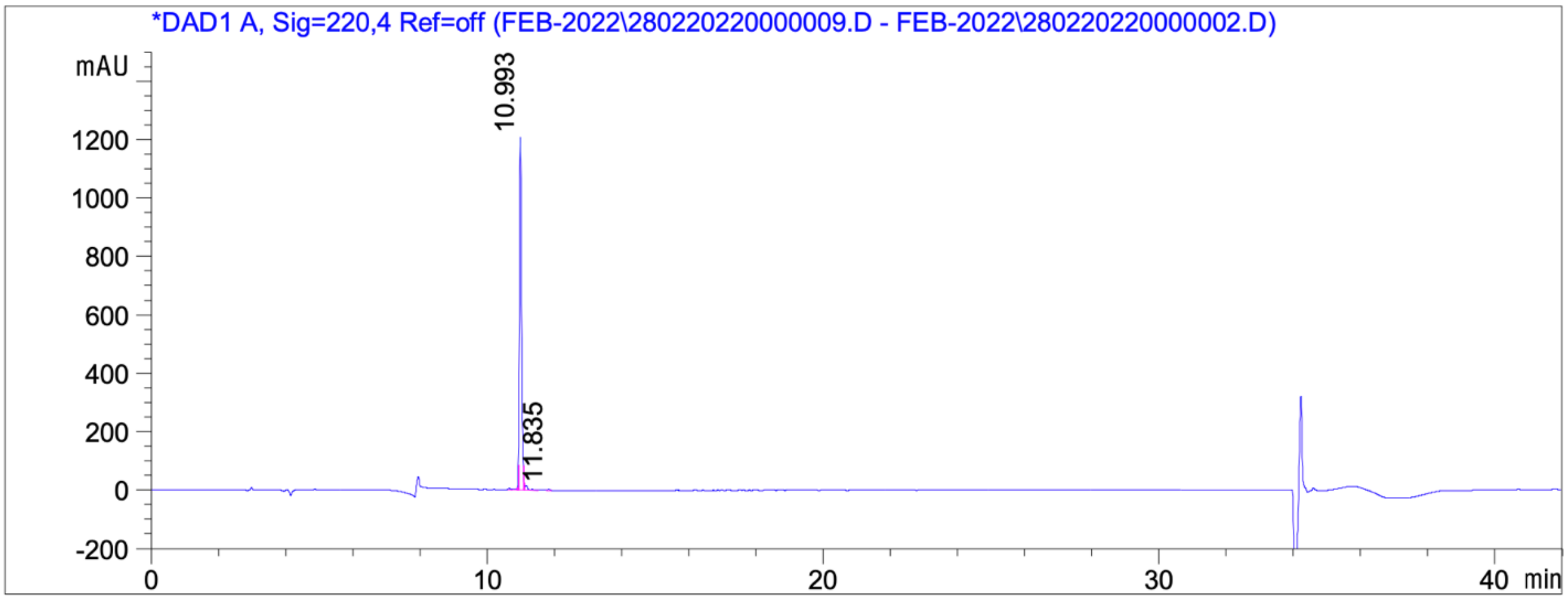

Mass spectrum

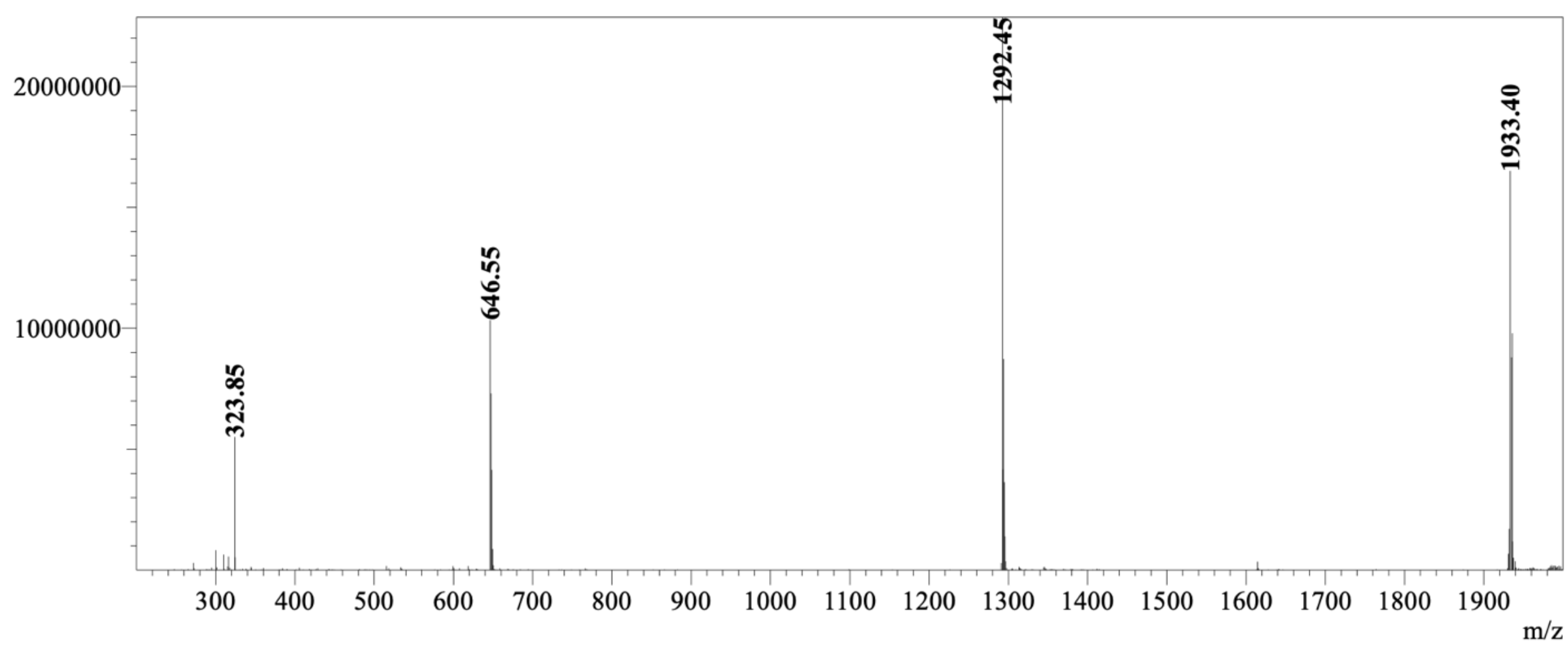

cyclo-SMDIKI

Analytical HPLC

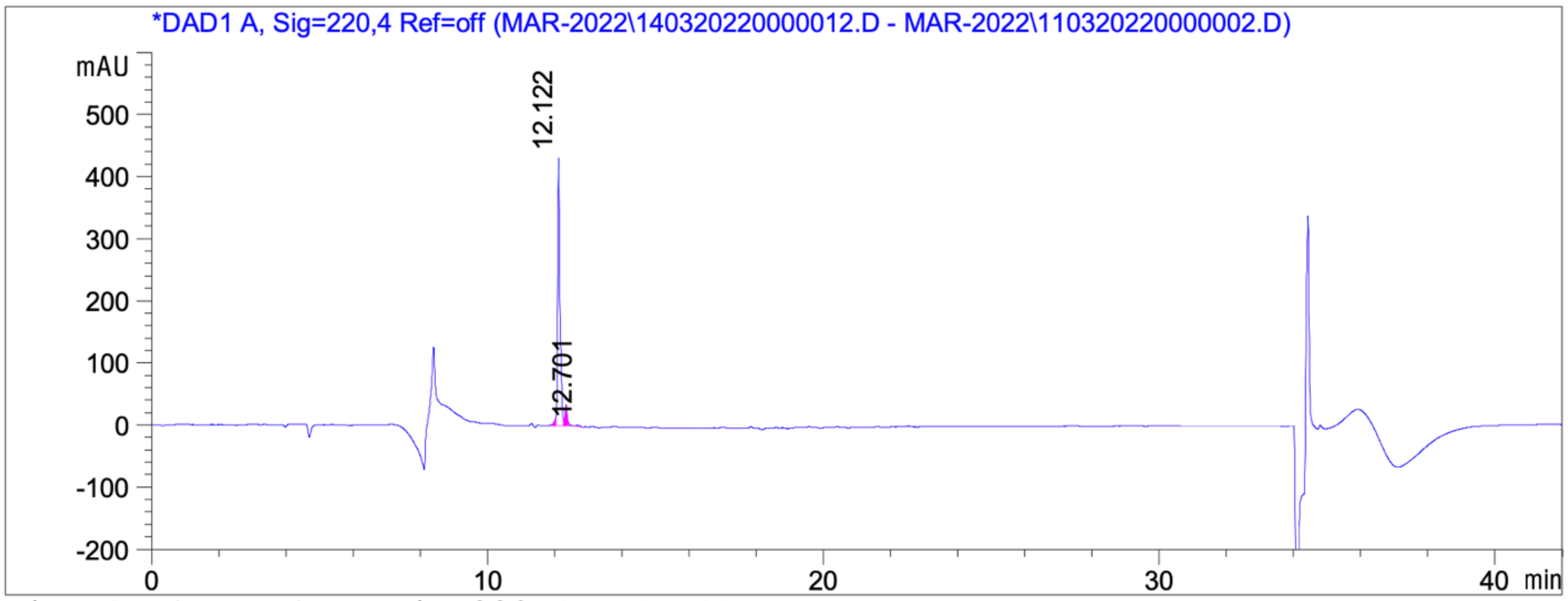

Mass spectrum

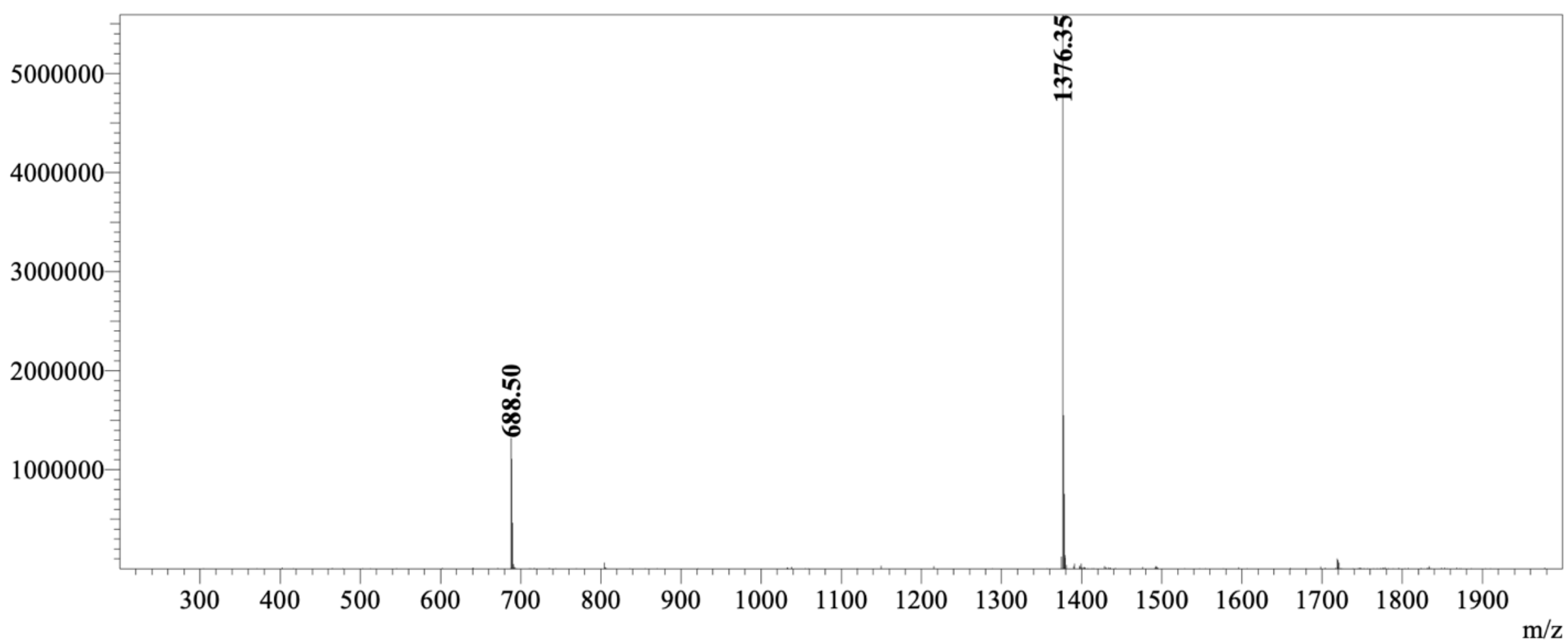

cyclo-SMDIKL

Analytical HPLC

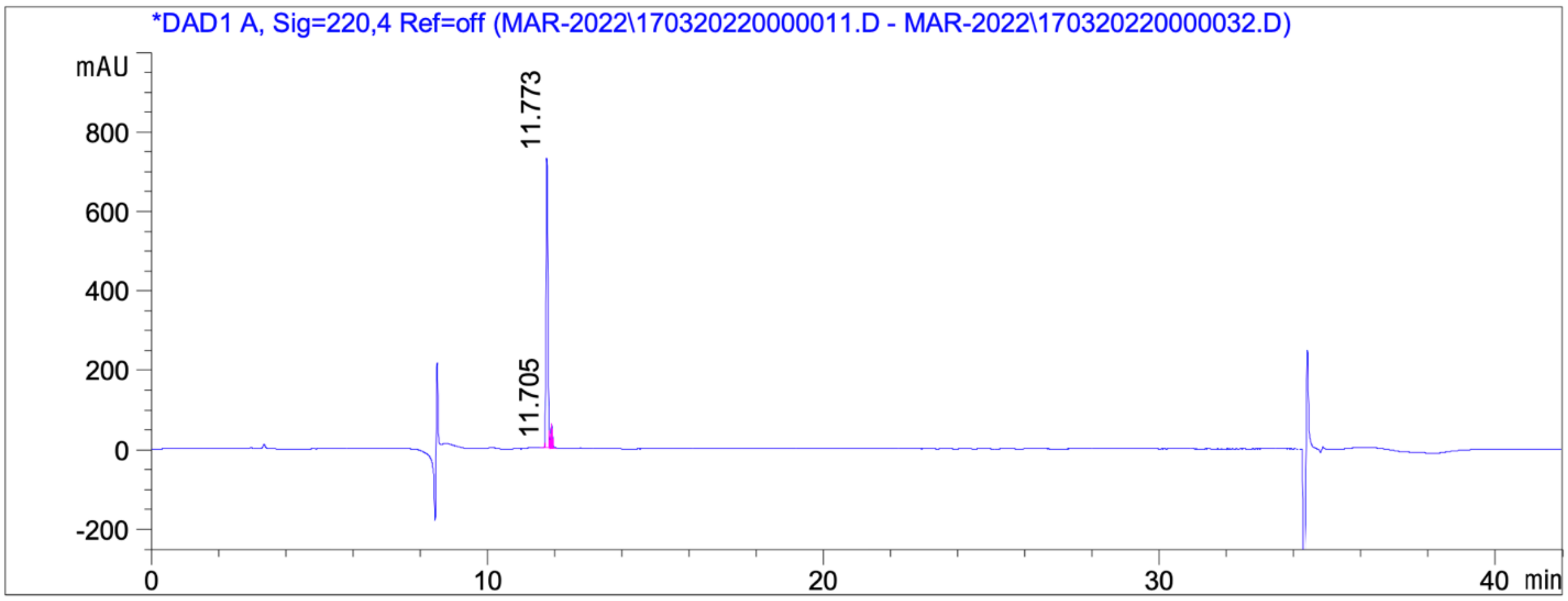

Mass spectrum

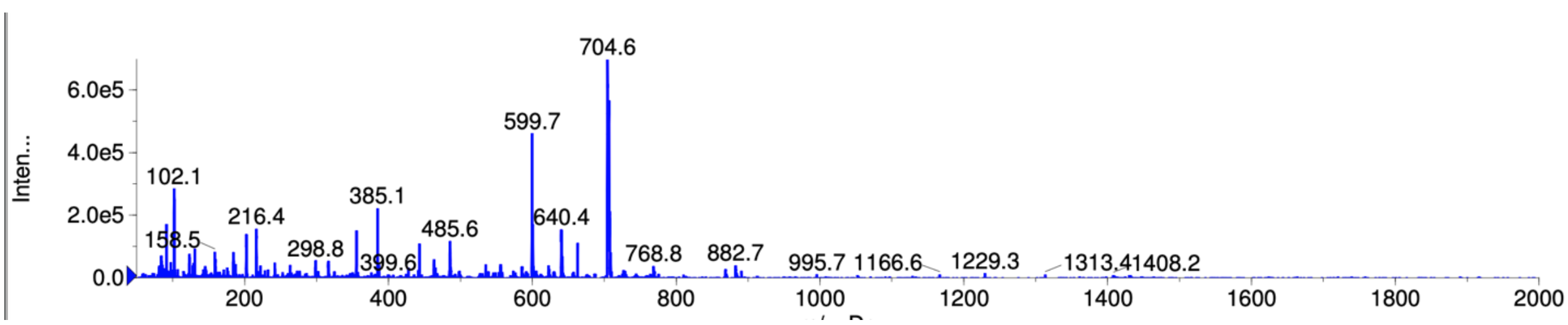

cyclo-SMDIKM

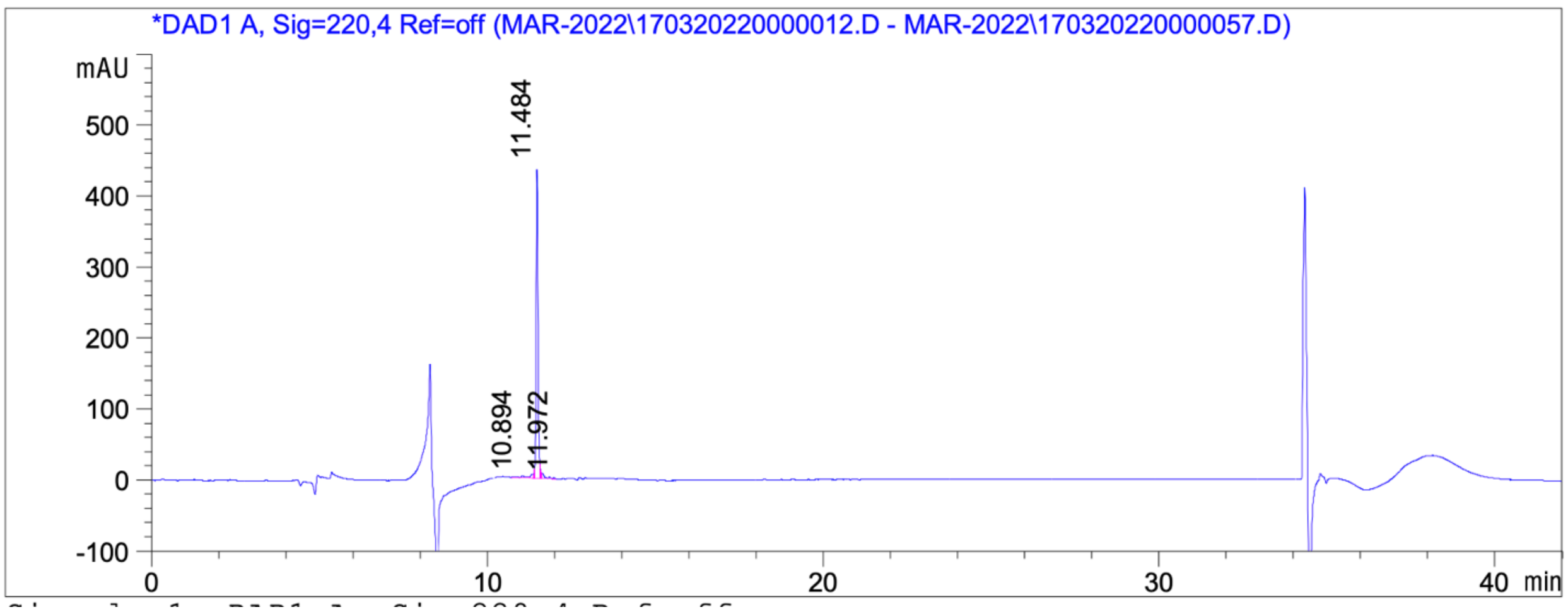

Analytical HPLC

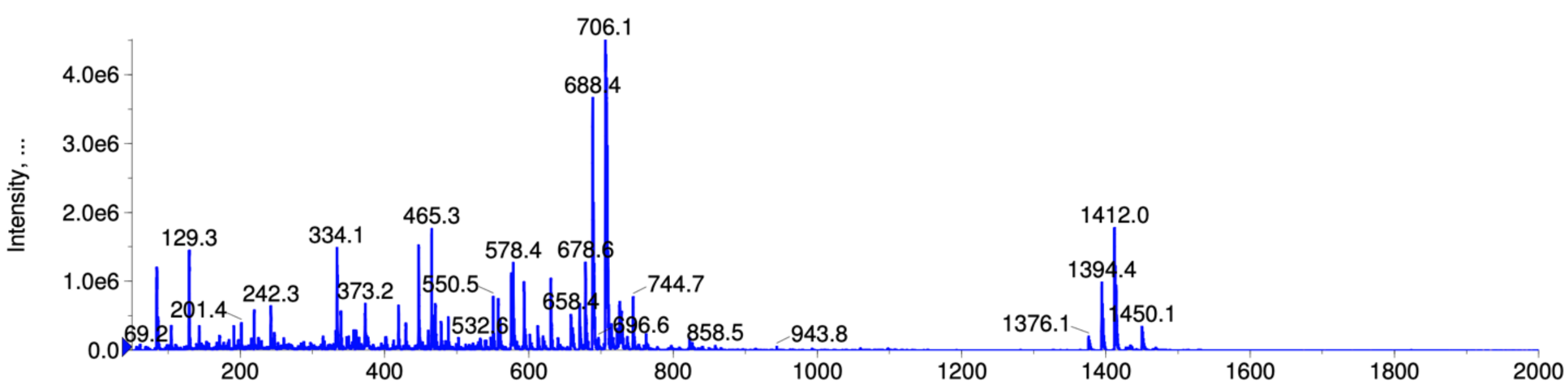

Mass spectrum

cyclo-SMDIKF

Analytical HPLC

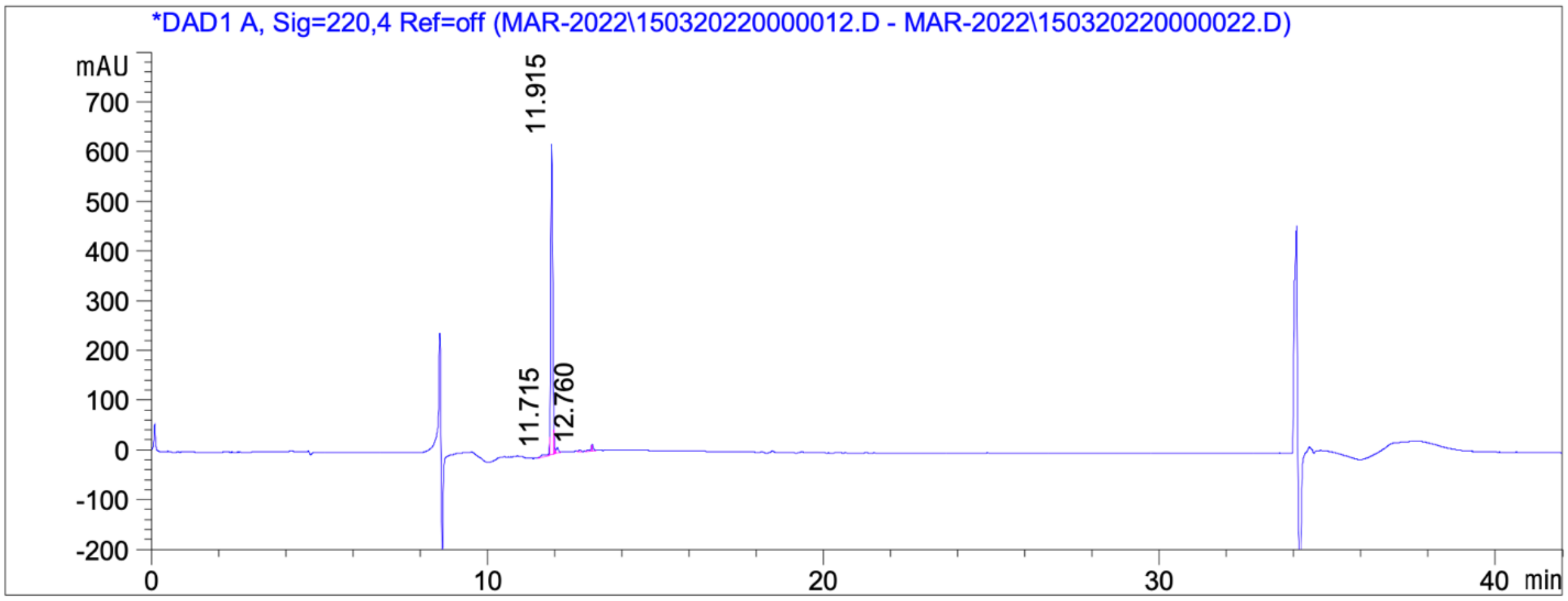

Mass spectrum

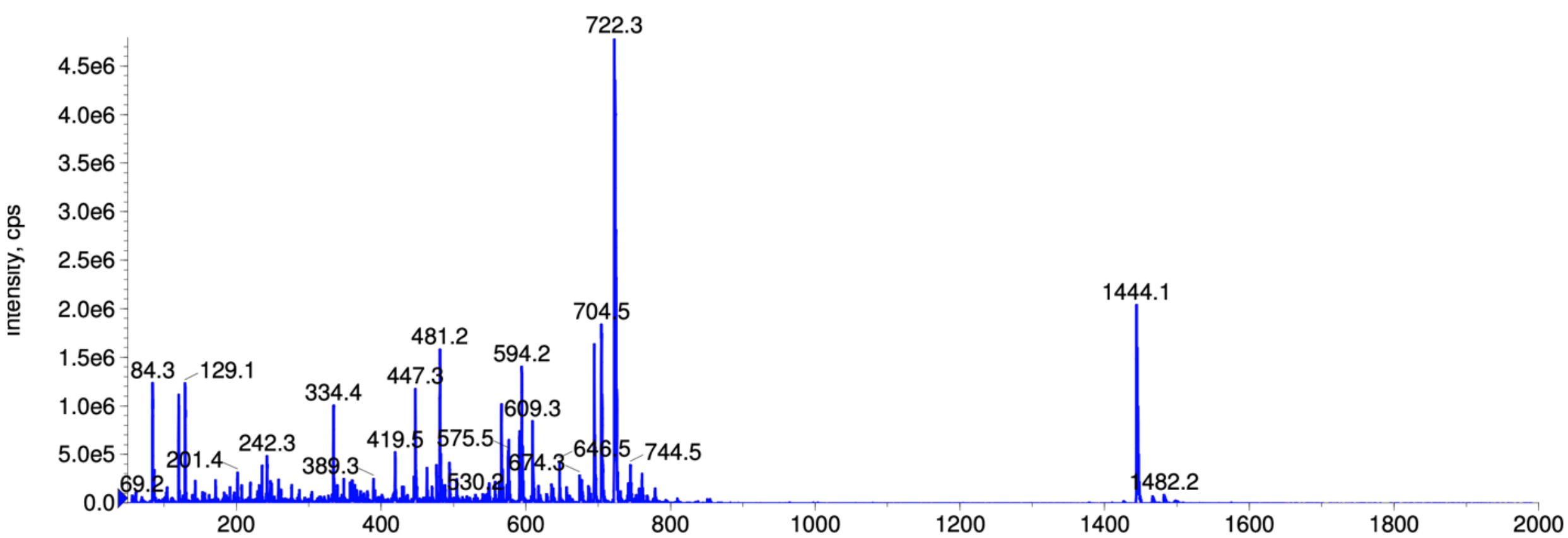

<u>*cyclo*-SMDIKW</u>

Analytical HPLC

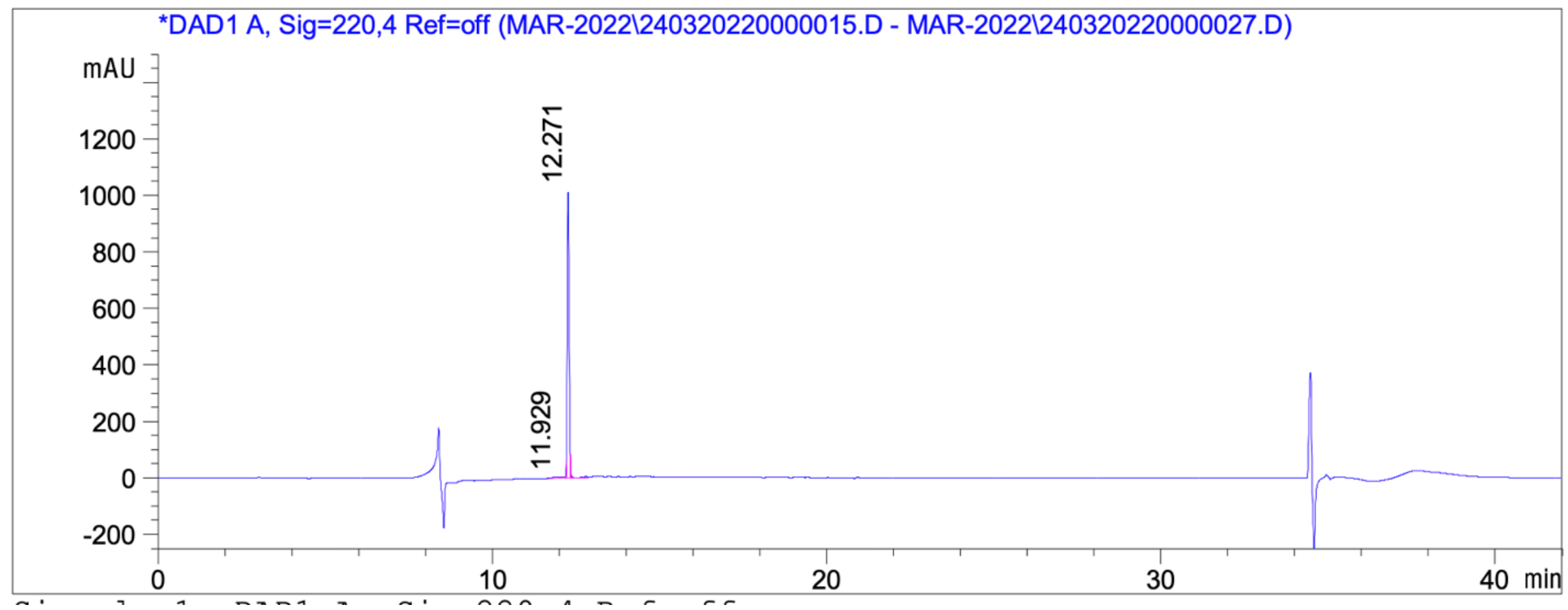

Mass spectrum

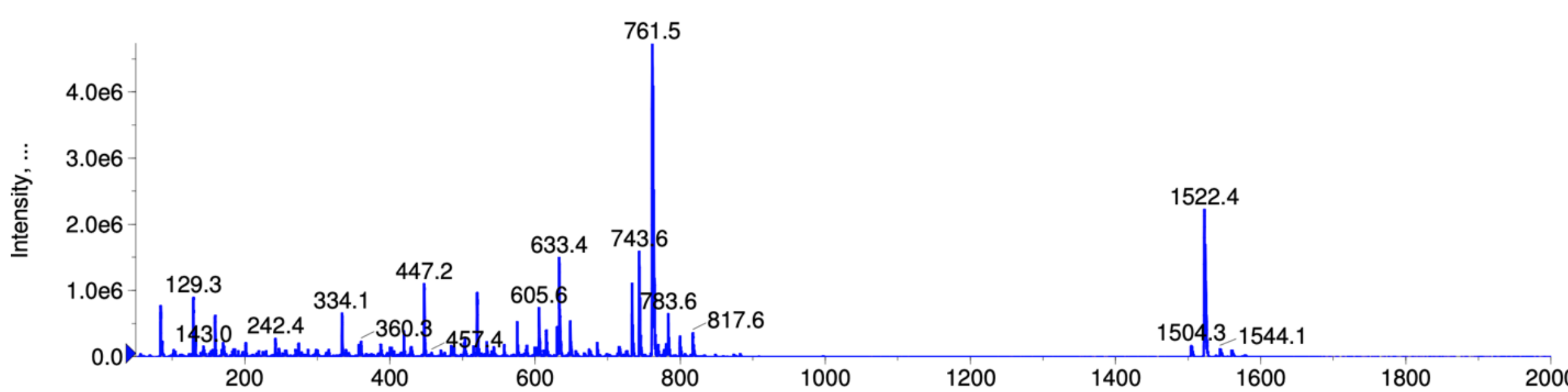

cyclo-SMDIKY

Analytical HPLC

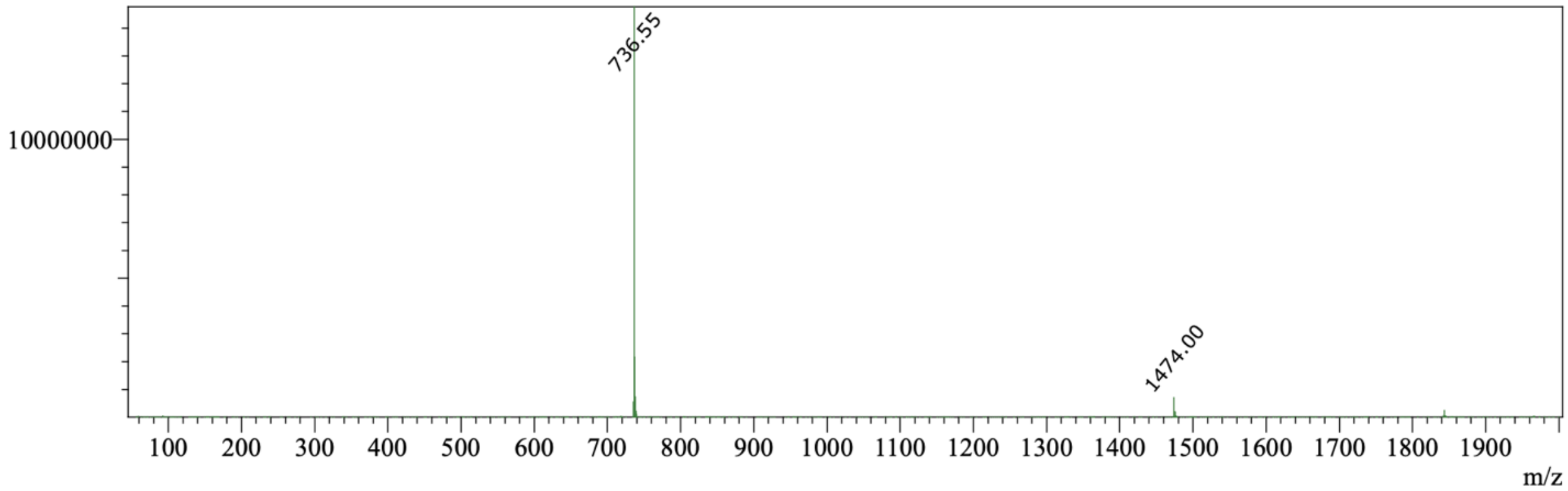

Mass spectrum

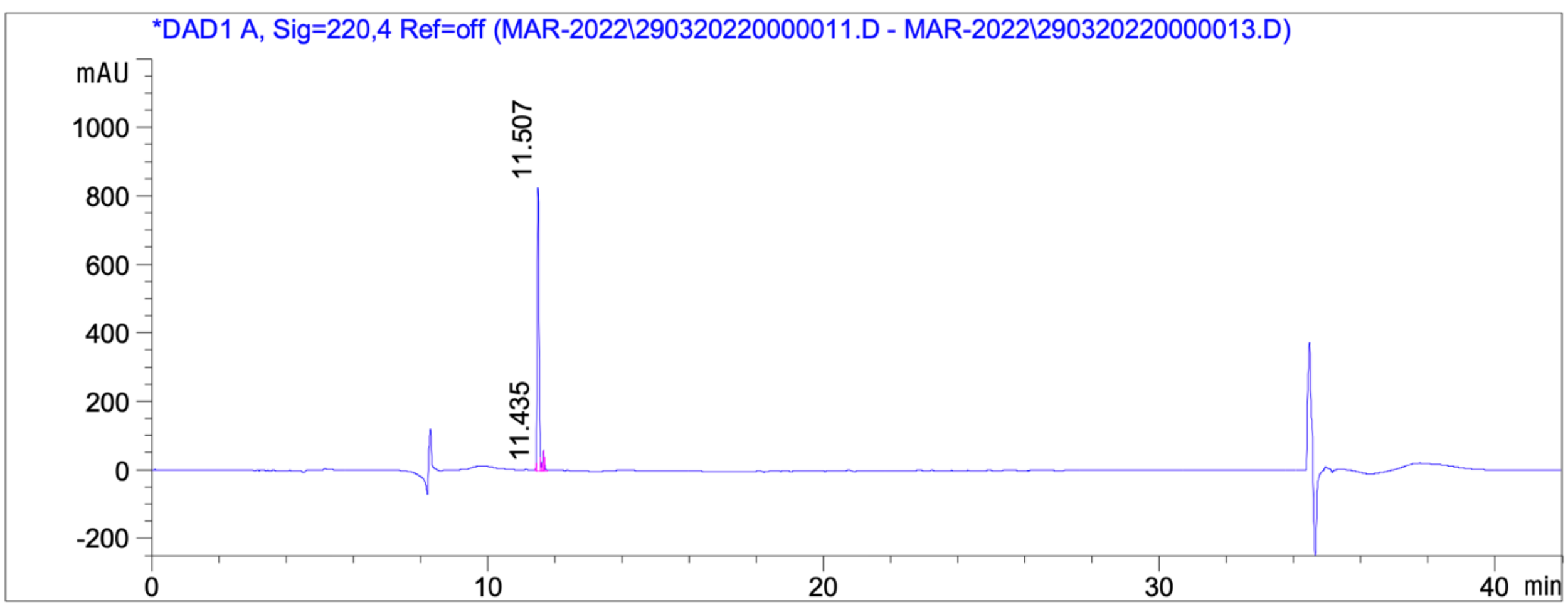

cyclo-SMDIKV

Analytical HPLC

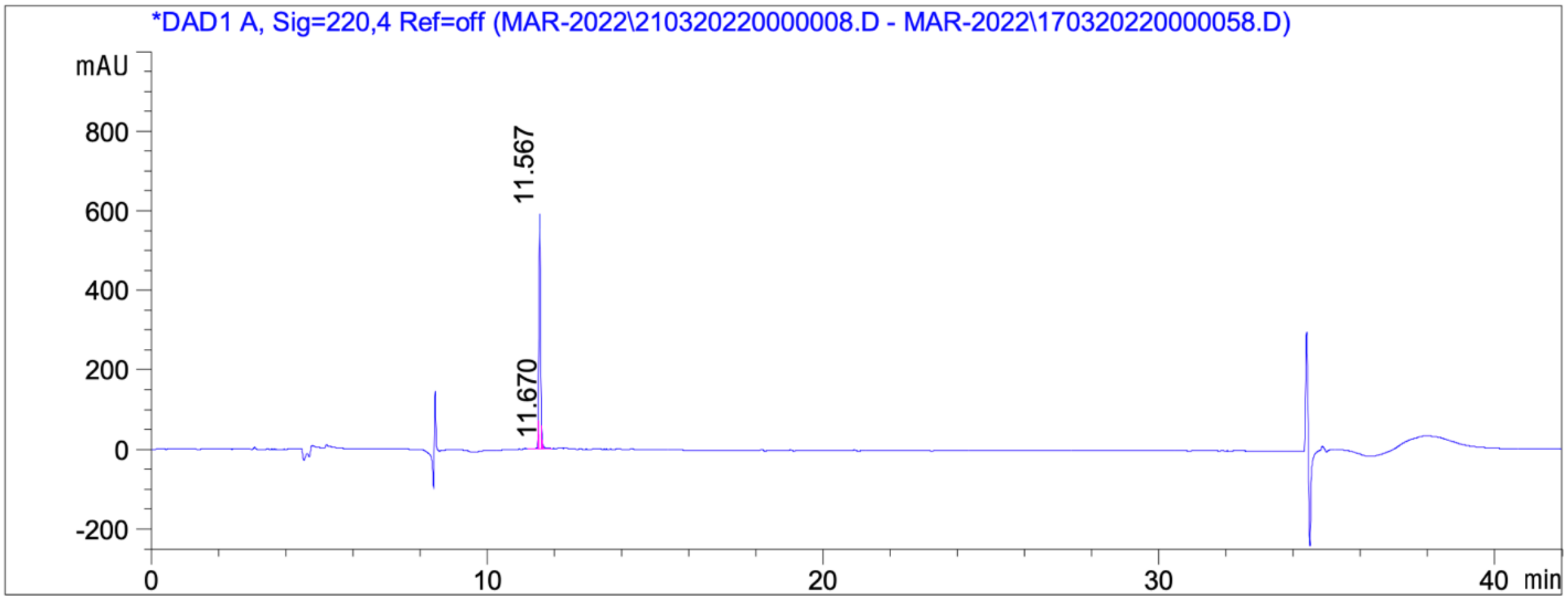

Mass spectrum

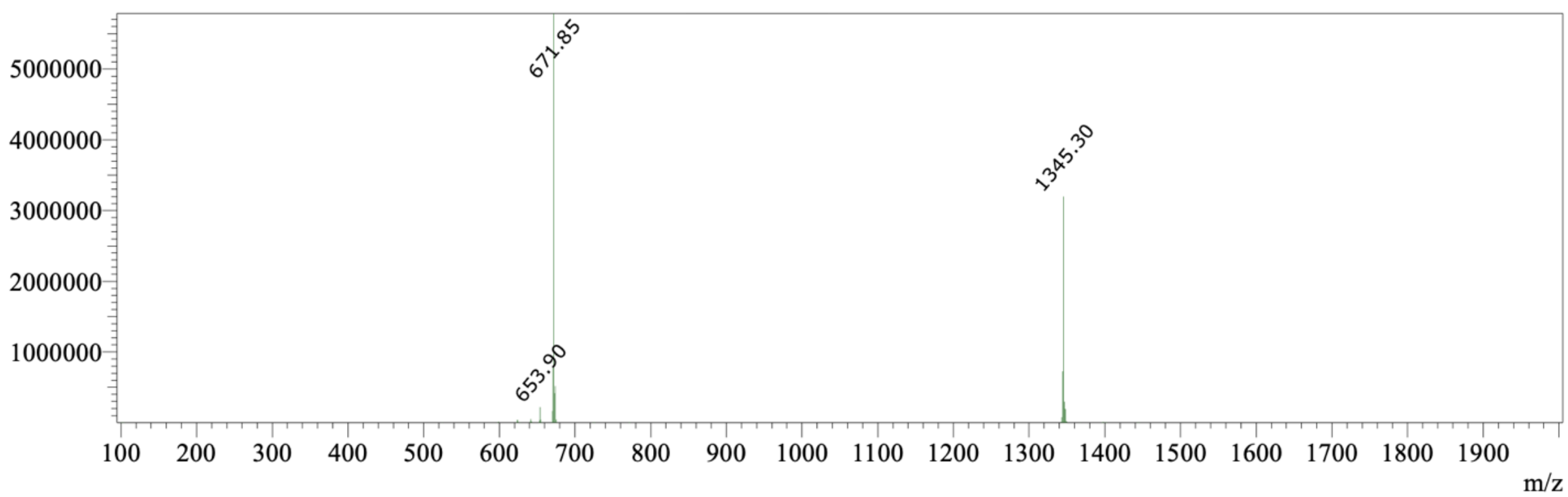

cyclo-SMDIKR

Analytical HPLC

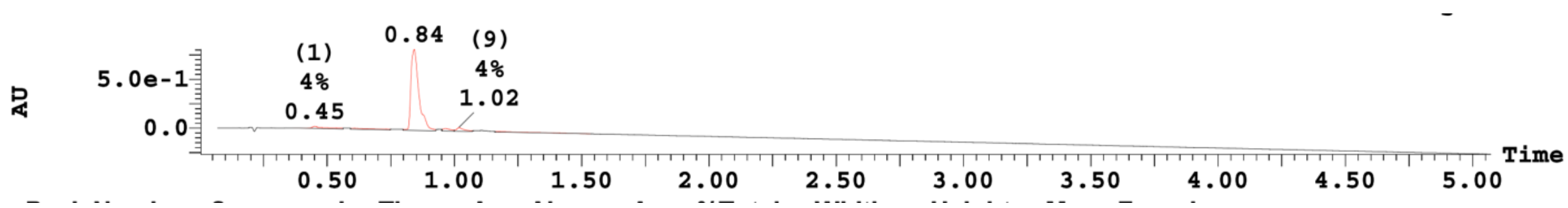

Mass spectrum

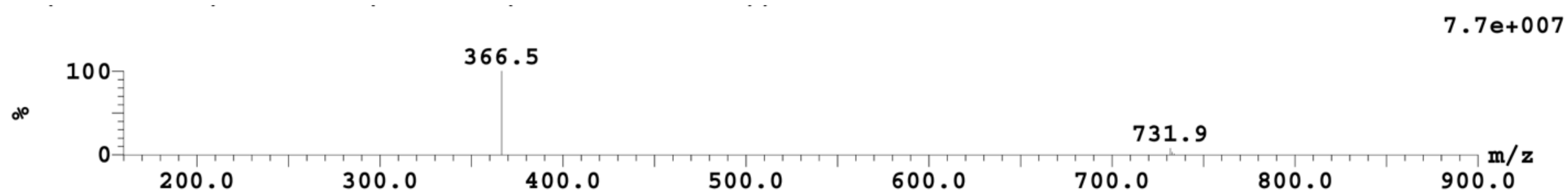

cyclo-SMDIKH

Analytical HPLC

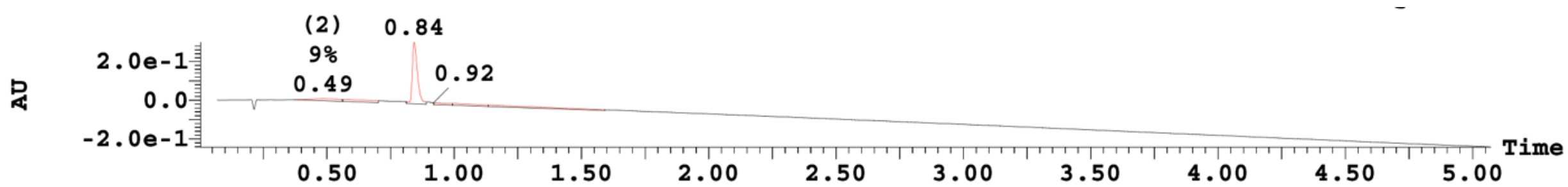

Mass spectrum

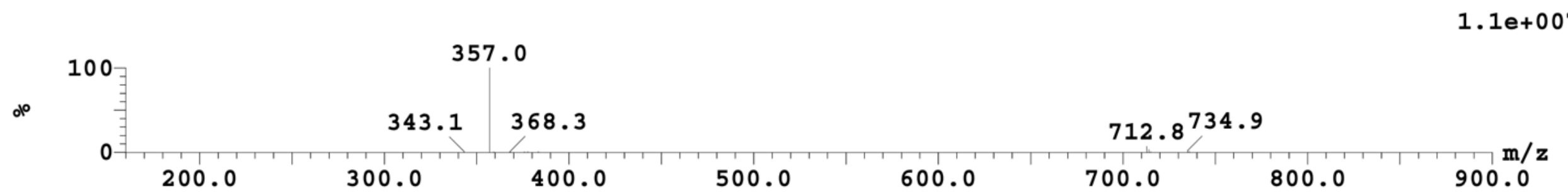

<u>*cyclo*-SIGMKD</u>

Analytical HPLC

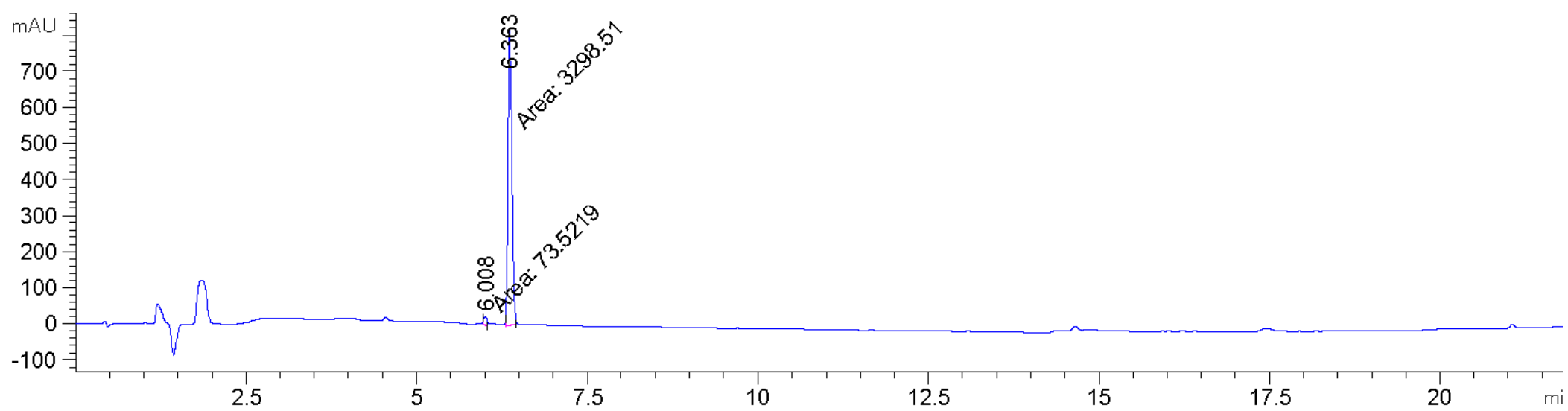

Mass spectrum

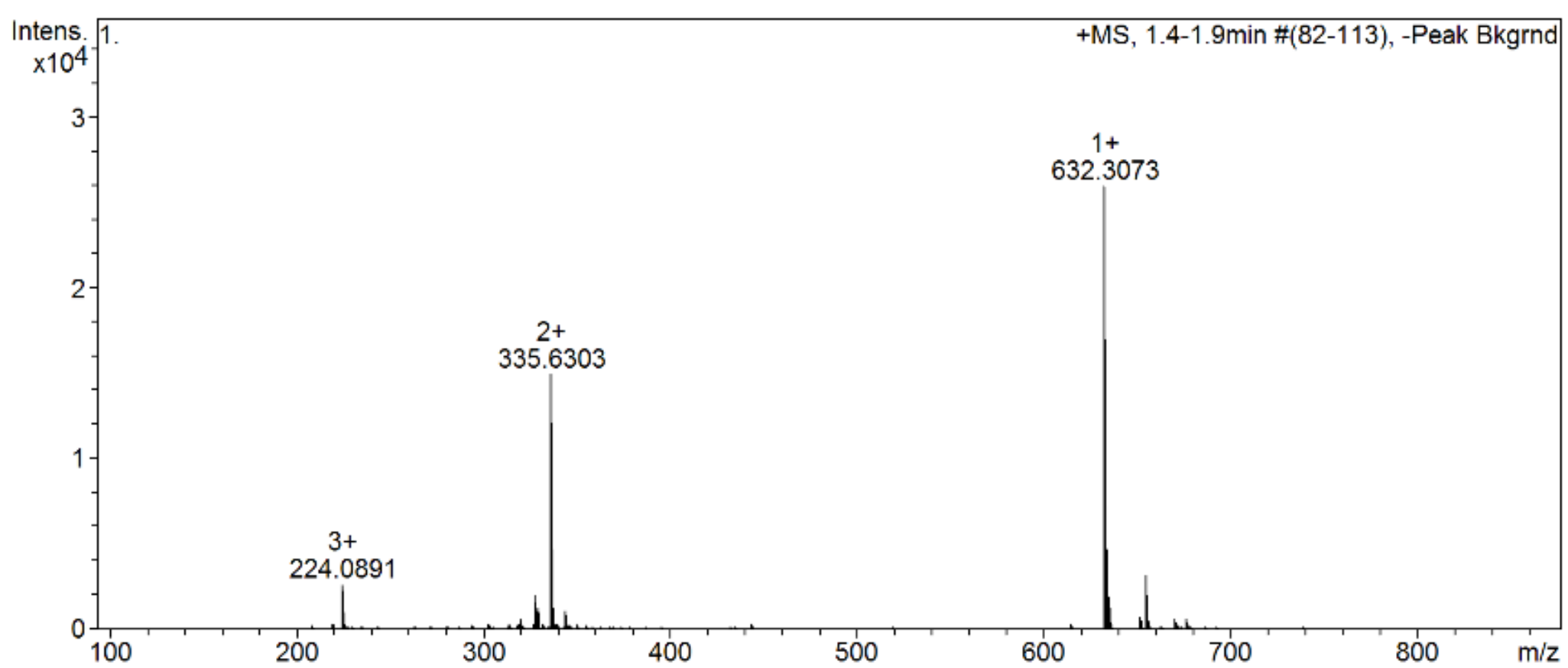

Meas. m/z	Formula	m/z	err [ppm]	err [mDa]	# Sigma	mSigma	rdb	e⁻ Conf	N-Rule
632.3073	C 25 H 43 N 11 Na O 5 S	632.3062	-1.9	-1.2	1	29.6	9.5	even	ok
	C 26 H 46 N 7 O 9 S	632.3072	-0.2	-0.1	2	30.6	7.5	even	ok
	C 28 H 51 N Na O 11 S	632.3075	0.3	0.2	3	34.4	3.5	even	ok
	C 26 H 39 N 15 Na O S	632.3075	0.2	0.2	4	40.2	14.5	even	ok
	C 27 H 42 N 11 O 5 S	632.3086	1.9	1.2	5	40.9	12.5	even	ok
	C 41 H 43 N 3 Na S	632.3070	-0.5	-0.3	6	93.7	21.5	even	ok

cyclo-SMDIKG click Probe

Mass Spectrum

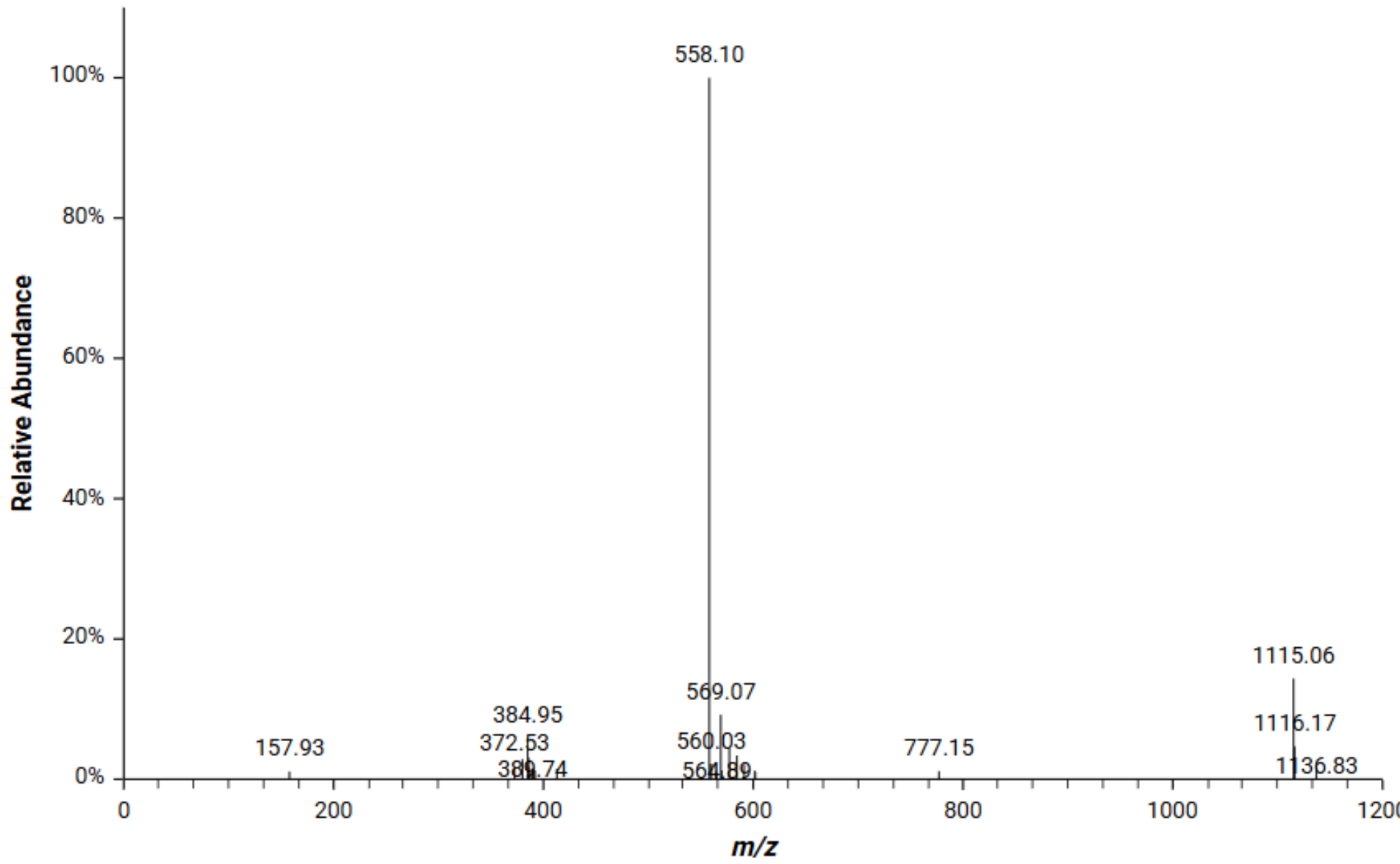

References

(1) Tavassoli, A.; Benkovic, S. J. Split-intein mediated circular ligation used in the synthesis of cyclic peptide libraries in E. coli. *Nat Protoc* **2007**, *2* (5), 1126-1133. DOI: 10.1038/nprot.2007.152 From NLM Medline.

(2) McDermott, A.; Windeln, L. M.; Valentine, J. S. D.; Baldassarre, L.; Foster, A. D.; Tavassoli, A. Next Generation SICLOPPS Screening for the Identification of Inhibitors of the HIF-1alpha/HIF-1beta Protein-Protein Interaction. *ACS Chem Biol* **2024**, *19* (10), 2232-2239. DOI: 10.1021/acschembio.4c00494 From NLM Medline.
